# Radiocarbon dating and isotope analysis on the purported Aurignacian skeletal remains from Fontana Nuova (Ragusa, Italy)

**DOI:** 10.1371/journal.pone.0213173

**Published:** 2019-03-20

**Authors:** Gianpiero Di Maida, Marcello A. Mannino, Ben Krause-Kyora, Theis Zetner Trolle Jensen, Sahra Talamo

**Affiliations:** 1 Graduate School Human Development in Landscapes, CAU Kiel, Kiel, Germany; 2 Neanderthal Museum, Mettmann, Germany; 3 Department of Archaeology and Heritage Studies, School of Culture and Society, Aarhus University, Højbjerg, Denmark; 4 Department of Human Evolution, Max Planck Institute for Evolutionary Anthropology, Leipzig, Germany; 5 Institute of Clinical Molecular Biology, CAU Kiel, Kiel, Germany; 6 Max Planck Institute for the Science of Human History, Jena, Germany; 7 Section for Evolutionary Genomics, Natural History Museum of Denmark, University of Copenhagen, Copenhagen, Denmark; 8 BioArCh, Department of Archaeology, University of York, York, United Kingdom; Universita degli Studi di Ferrara, ITALY

## Abstract

Proving voyaging at sea by Palaeolithic humans is a difficult archaeological task, even for short distances. In the Mediterranean, a commonly accepted sea crossing is that from the Italian Peninsula to Sicily by anatomically modern humans, purportedly of the Aurignacian culture. This claim, however, was only supported by the typological attribution to the Aurignacian of the lithic industries from the insular site of Fontana Nuova. AMS radiocarbon dating undertaken as part of our research shows that the faunal remains, previously considered Aurignacian, actually date to the Holocene. Absolute dating on dentinal collagen also attributes the human teeth from the site to the early Holocene, although we were unable to obtain ancient DNA to evaluate their ancestry. Ten radiocarbon dates on human and other taxa are comprised between 9910–9700 cal. BP and 8600–8480 cal. BP, indicating that Fontana Nuova was occupied by Mesolithic and not Aurignacian hunter-gatherers. Only a new study of the lithic assemblage could establish if the material from Fontana Nuova is a mixed collection that includes both late Upper Palaeolithic (Epigravettian) and Mesolithic artefacts, as can be suggested by taking into account both the results of our study and of the most recent reinterpretation of the lithics. Nevertheless, this research suggests that the notion that Aurignacian groups were present in Sicily should now be revised. Another outcome of our study is that we found that three specimens, attributed on grounds both of morphological and ZooMS identifications to *Cervus elaphus*, had δ^13^C values significantly higher than any available for such species in Europe.

## Introduction

The question of voyaging at sea in the Mediterranean during the Palaeolithic is still open, even when it comes to the Upper Palaeolithic [1]. One of the few claims for a sea crossing around the time of the first arrival of anatomically modern humans to Europe, coinciding with the inception of the Aurignacian culture, is that made on the basis of the Sicilian site of Riparo di Fontana Nuova. In his seminal book titled *The Making of the Middle Sea*, Broodbank [2] states that this claim possesses “one advantage over earlier claims of island occupation in the Mediterranean: it indisputably happened”. This view is shared by most authors that have dealt in detail with the issues of prehistoric voyaging at sea in the Mediterranean Sea [3–4]. The cultural and chronological attributions of the site to the Aurignacian, however, solely hinge on chrono-typological considerations resulting from the study of the lithic assemblage recovered during unsystematic excavations [5–12]. A more recent revision of the lithic assemblage from Fontana Nuova has typologically attributed it to the closing stages of the Upper Palaeolithic, specifically to the Final (or Late) Epigravettian [13]. None of the above-mentioned studies included absolute dating, which we have undertaken as part of the research presented here.

### The site and its lithic assemblage

**Fig 1:**
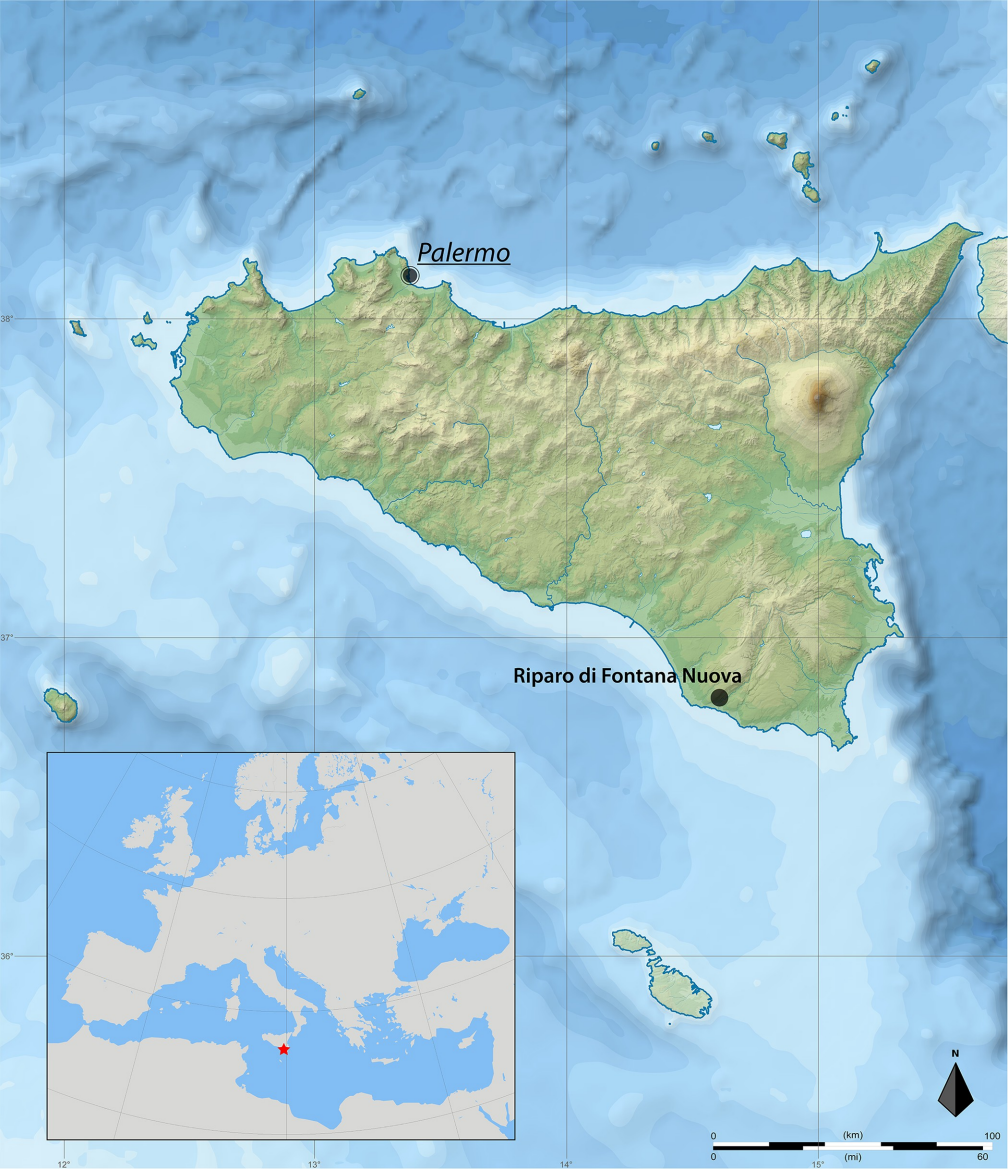
Location of Riparo di Fontana Nuova in south-eastern Sicily. Maps modified from: https://commons.wikimedia.org/wiki/File:Regione_Siciliana_topographic_map-blank.svg# and https://commons.wikimedia.org/wiki/File:Blank_map_of_Europe_(polar_stereographic_projection)_cropped.svg.

The first archaeological activity at the site of Riparo di Fontana Nuova (Fig 1) was the unsystematic excavation conducted by a local nobleman, Vincenzo Grimaldi di Calamenzana, sometime before January 1914. In that month, in fact, he donated the lithic assemblage to the Archaeological Museum in Syracuse, having reburied the skeletal remains [14]. Following World War II, after Luigi Bernabò Brea was appointed ‘Soprintendente delle Antichità per la Sicilia Orientale’, the collections of the Museum were reorganized and, in the process, the boxes containing the lithic material were retrieved. This ‘re-discovery’ pushed Bernabò Brea to search for the site and in 1949 he was able to locate the spoil heap of Baron Calamenzana’s excavation, retrieving the unstratified skeletal finds as a record of what had been present at the site. During his visit, Bernabò Brea was also able to observe the remnants of the original stratigraphic section at Fontana Nuova, observing that it consisted of three distinct layers and concluding that the prehistoric material from the spoil heap must all have originated from the discrete dark middle layer containing charcoal and bone fragments. Bernabò Brea [14] published the first description of the material, leaving open the issue of its exact chronology, attributing the assemblage broadly to the Upper Palaeolithic. The importance of Fontana Nuova for the study of the Sicilian Upper Palaeolithic started with the first attribution of its lithic assemblage to the Aurignacian by Laplace in his seminal research on the Italian lithic assemblages [5]. The French archaeologist observed the peculiarity of the complex (“plus aurignacoïde qu’aurignacien”) and justified the absence of diagnostic bone tools by suggesting that the material culture was compatible with a relatively evolved phase of the Aurignacian (“en l’absence de toute forme osseuse caractéristique, pouvoir être attribué à une phase relativement évoluée de l’Aurignacien”).

The most detailed analysis of the lithic assemblage recovered at Riparo di Fontana Nuova is the one by Gioia [7], reviewed in 1996 by the same researcher [12], using a statistical analysis based on the typological list proposed by de Sonneville-Bordes & Perrot [15]. The total number of pieces examined was 212 in the first paper and 224 in the second, given that an additional 12 lithics were found among the faunal remains. The lithic assemblage is composed of 60.7% retouched tools, 33.5% by unretouched blades and flakes, and 5.8% cores. Overall, the lithic industry is markedly laminar and endscrapers are the main typological group (40.4%), which purportedly includes several Aurignacian types, such as keeled endscrapers, nosed endscrapers, endscrapers on an Aurignacian blade [12]. The assemblage has also been reported to include blades with scalar Aurignacian retouch and strangulated blades. These interpretations have led Chilardi et al. [12] to confirm the broad scheme proposed by Laplace [5]. However, Gioia [7] disagreed with the hypothesis put forward by Laplace [5] according to which the lithic industries from Fontana Nuova belonged to a peripheral and regional kind of Aurignacian. In fact, by comparing the techno-complex with some of the most typical industries of the Périgord (e.g. Caminade Ouest, Abri Cellier, La Ferrassie) and the few known for the Italian Peninsula, Gioia [7] placed it in phase I of the Aurignacian and connected it directly with the techno-complexes of human groups in France, hypothesizing a rapid movement of this culture from there to Italy [12]. The purported Aurignacian chronology of Fontana Nuova was also supported by arguing that its faunal assemblage differed from that of other Upper Palaeolithic (but in that case Epigravettian) sites on Sicily, which all post-date the Last Glacial Maximum (hereafter LGM). Chilardi et al. [12] argued that the absence of *Equus hydruntinus*, an equid that arrived on the island from southern Italy at the time of its land-bridge connection with Calabria [16], was proof that Riparo di Fontana Nuova was occupied before the LGM. The investigations on the different find categories conducted by Chilardi et al. [12] also concluded that the lithic assemblage, faunal and human remains all suggest that the site was not intensively or repeatedly occupied much over time, and was probably a short-term encampment.

The Aurignacian attributions of the lithic industries from Fontana Nuova were justified by the absence of microliths, backed points and blades (recurrent elements in Sicilian Epigravettian and Mesolithic lithic complexes), and by the presence of middle and large size pieces [12], as well as of what are regarded as diagnostic Aurignacian blades and scrapers. The first to doubt the attribution to the Aurignacian was Palma di Cesnola [9,10], not only because of the features of the assemblage, but also because of the patchy distribution of Aurignacian sites in southern Italy. Originally, Martini [17] was more inclined to accept the first attribution by Laplace [5], agreeing that this lithic industry was a regional and peripheral version of the classic French Aurignacian. In a more recent paper though [13], the Aurignacian attribution was rejected for the first time in favour of a much younger chronology, due to the very close resemblance of the lithic complex from Fontana Nuova with that of Grotta delle Uccerie (Favignana), which has been dated to 13191±120 (LTL1517A) and 12933±75 BP (LTL1518A) [13]. Martini and colleagues have pointed out that the Fontana Nuova complex is missing both the stylistic and structural traits typical of the Italian Aurignacian and that the lithics that have been interpreted as Aurignacoid elements (e.g. sub-carinated scrapers, blades with sinuous margins) are indeed elements compatible with the early (phase 1) Late Epigravettian of Sicily.

The chronological attributions of the lithic assemblage from Riparo di Fontana Nuova have all simply been based on chrono-typological criteria and none of them have been supported by absolute dating of the faunal and human remains, which have been so far considered coeval with the lithic assemblage (e.g. [12]). The main contribution of this research is to provide absolute dates on the skeletal remains.

## Materials and methods

A total of 25 human (repository numbers 10207, 10210, 10211) and other faunal remains (no repository numbers available), listed in detail in the Supporting Information (S1 Table), were sampled for the present study at the Museo Archeologico Regionale ‘Paolo Orsi’ in Syracuse (Italy), where they remain available to other researchers. All necessary permits were obtained for the described study, which complied with all relevant regulations. As the attempt to extract collagen by Chilardi et al. [12] had failed, we exercised great caution in selecting the specimens to pretreat. It should be noted (as can be seen in the photos provided in the Supporting Information) that the preservation is quite homogeneous and that none of the specimens bears traces of consolidants or solvents. A detailed taphonomic study of the small faunal assemblage has not been undertaken, because according to Chilardi et al. [12] the bone surfaces are poorly preserved. Nevertheless, many of the bones in the assemblage bear traces of burning and green fractures, resulting from dismemberment and extensive processing of the carcasses.

### Isotopes analyses and AMS radiocarbon dating

Bone pretreatment methods for isotope analyses and AMS radiocarbon dating are those established by Talamo and Richards [18] and the extractions were conducted at the Max Planck Institute for Evolutionary Anthropology in Leipzig (MPI-EVA, lab Code R-EVA).

The two human teeth were micro-CT scanned before sampling at the Department of Human Evolution of the above-mentioned institution. The three-dimensional digital images resulting from the scans are shown in the Supporting Information (S1 and S2 Figs).

The outer surface of the samples was first cleaned with a shot blaster and then 500mg of bone was taken. The samples were decalcified in 0.5M HCl at room temperature until no CO_2_ effervescence was observed, usually for about 4 hours. 0.1M NaOH was added for 30 minutes to remove humics. The NaOH step was followed by a final 0.5M HCl step for 15 minutes. The resulting solid was gelatinized following Longin [19] at pH3 in a heater block at 75°C for 20h. The gelatine was then filtered in an Eeze-Filter (Elkay Laboratory Products (UK) Ltd.) to remove small (>80 μm) particles. The gelatine was then ultrafiltered with Sartorius “Vivaspin Turbo” 30 KDa ultrafilters [20]. Prior to use, the filters were cleaned to remove carbon containing humectants [21]. The samples were lyophilized for 48 hours.

The isotope analyses were performed on a Thermo Finnigan Delta V Advantage Isotope Ratio Mass Spectrometer (IRMS) coupled to a Flash 2000 EA. Stable carbon isotope ratios are expressed relative to the VPDB (Vienna Pee Dee Belemnite) standard and stable nitrogen isotope ratios relative to AIR (Atmospheric N_2_). The analytical error is 0.2‰ (1σ). Collagen from all 10 samples that yielded well-preserved extracts were sent to the Klaus Tschira Laboratory of the Curt-Engelhorn-Zentrum Archaeometrie in Mannheim (MAMS) for AMS radiocarbon dating, which was performed using the Mini Radiocarbon Dating System (MICADAS) [22]. The results of the AMS radiocarbon dating are reported in Table 1.

**Table 1:** Isotopic, elemental analyses and radiocarbon dates.

MPI-laboratory number	species	element	δ^13^C (‰)	δ^15^N (‰)	%C	%N	C:N	% collagen yield	AMS radiocarbon laboratory code	^14^C date (BP)
R-EVA-1862	*Cervus elaphus*	humerus	-16.1	6.0	40.7	14.6	3.3	4.3	MAMS-30404	8285 ± 20
R-EVA 1865	*Cervus elaphus*	scafocuboid	-19.8	5.5	35.6	12.8	3.2	4.0	MAMS-30405	8699 ± 22
R-EVA-1866	*Cervus elaphus*	radius	-21.0	5.4	34.8	12.5	3.3	2.1	MAMS-30406	8701 ± 22
R-EVA-1871	*Sus scrofa*	metacarpal III	-20.6	4.9	36.5	13.0	3.3	5.1	MAMS-30407	7775 ± 20
R-EVA-1877	*Cervus elaphus*	tibia	-15.9	6.2	38.8	13.9	3.3	3.5	MAMS-30409	8680 ± 21
R-EVA-1878	*Cervus elaphus*	tibia	-20.3	5.9	40.3	14.5	3.2	4.6	MAMS-30410	8597 ± 22
R-EVA-1880	*Cervus elaphus*	tibia	-16.8	6.1	39.4	14.0	3.3	2.5	MAMS-30411	8793 ± 22
R-EVA-1881	*Cervus elaphus*	phalanx I	-19.5	6.2	37.6	13.6	3.2	5.4	MAMS-30412	8720 ± 22
R-EVA-1895	*Homo sapiens*	molar	-19.3	11.9	42.2	15.2	3.2	4.1	MAMS-30660	8675 ± 25
R-EVA-1896	*Homo sapiens*	premolar	-19.4	12.0	40.2	14.0	3.3	2.2	MAMS-30663	8658 ± 25

Carbon (δ^13^C) and nitrogen (δ^15^N) isotope values, elemental compositions, carbon-to-nitrogen ratios and AMS radiocarbon dates of the collagen from well-preserved skeletal remains recovered at Riparo di Fontana Nuova. The *C*. *elaphus* tibia belong to three clearly different individuals, given that they are all right side specimens. The state of preservation and size of the *C*. *elaphus* humerus suggest tentatively that a fourth individual may be represented in our isotopic dataset. The samples were pretreated at the Max Planck Institute for Evolutionary Anthropology (Leipzig) and dated at the Klaus Tschira Laboratory of the Curt-Engelhorn-Zentrum Archaeometrie in Mannheim (MAMS).

The animal remains sampled belong to *Cervus elaphus* (n = 16), *Bos primigenius* (n = 3) and *Sus scrofa* (n = 3), as listed in greater detail in the Supporting Information (S1 Table). Of these, only 8 specimens yielded extracts, all of which can be considered well-preserved collagen (Table 1), according to the quality criteria proposed by van Klinken [23]. All well-preserved extracts from the fauna were used for ZooMS (Zooarchaeology by Mass Spectrometry), according to the methods described below. The three human (*Homo sapiens*) specimens sampled include a cranial (parietal) fragment and two maxillary teeth (a premolar and a molar). According to Chilardi et al. [12], all human skeletal remains from Riparo di Fontana Nuova may have belonged to a single adult individual. The cranial fragment (R-EVA 1883) did not yield an extract. The teeth (R-EVA 1895 and R-EVA 1896) have extracts compatible with well-preserved collagen according to the criteria proposed by van Klinken [23], given that their elemental (%C, %N) and isotopic (δ^13^, δ^15^N) compositions, C:N ratios and yields fall within biogenic ranges (Table 1).

### aDNA

Ancient DNA (aDNA) extractions and pre-PCR steps were carried out in clean room facilities dedicated to aDNA research at the Ancient DNA Laboratory of the Max Planck Institute for the Science of Human History (MPI SHH) in Jena. Tooth fragments of samples R-EVA 1895 and R-EVA 1896 were used for the analyses. All procedures followed the guidelines on contamination control in aDNA studies [24,25]. The teeth were UV-radiated to remove potential contaminants prior to drilling. Fifty milligrams of powder were used for extraction following a silica-based protocol [26]. Negative controls were included at all steps.

Double-stranded DNA sequencing libraries (UDGhalf) were prepared for each sample according to an established protocol for multiplex high-throughput sequencing [27]. Sample-specific indices were added to both library adapters via amplification with two index primers. The libraries were sequenced on 1/50 of a lane on the HiSeq 3000 (2x75 bp) at the MPI SHH in Jena, using the HiSeq v4 chemistry and the manufacturer’s protocol for multiplex sequencing.

The adapter sequences were removed and overlapping paired-end reads were merged with ClipAndMerge, which is a module of the EAGER pipeline [28]. Mapping of the adapter-clipped and merged FASTQ files to the human reference genome hg38 was done using BWA [29] using a reduced mapping stringency of “-n 0.01” and the mapping quality parameter “q 30”.

### ZooMS

Eight faunal remains, sampled for AMS and isotope analyses were selected for ZooMS (Zooarchaeology by Mass Spectrometry) for species identification, to further strengthen the argument for intrapecies differences in the isotopic composition of *C*. *elaphus*.

Peptide extraction for ZooMS was carried out at Centre for GeoGenetics, Natural History Museum, University of Copenhagen, Denmark and MALDI-TOF-MS (matrix-assisted laser desorption/ionization time-of-flight mass spectrometry) analysis was subsequently done at the Centre for Excellence in Proteomics at the University of York (United Kingdom).

An average of 16.1 mg of bone was cut off of the selected bone samples using a circular saw-blade attached to a Dremel saw. To circumvent cross-sample contamination the blade was rinsed in 10% bleach and 70% ethanol in between samples.

Prior to protein extraction, each bone chip was incubated in 100 μL of 50 mM ammonium bicarbonate solution (NH_4_HCO_3_) pH 8.0 (AmBic) for 16 hours at ambient temperature. The samples were then vortexed for 15 seconds and centrifuged at 13,000 rpm for 1 min, and the supernatant discarded. To remove potential humic acids (which interfere with the MALDI-TOF-MS) 100 μL of NaOH was added and the samples were centrifuged at 13,000 rpm for 1 min, the supernatants were discarded and the bone fragments were washed three times in AmBic to neutralize pH. After the final wash the samples were incubated in 100 μL AmBic at 65°C for 60 minutes to gelatinize the collagen. 50 μL of each supernatant was transferred into a new 1.5 mL eppendorf tube (labelled “extraction”). After cooling to ambient temperature, 1 μL of sequence grade Trypsin (Promega) was added to each of the extractions which were then incubated at 37°C for 16 hours. Following trypsin digestion, the extractions were centrifuged at 13,000 rpm for 1 min and 1 μL of 5% Trifluoroacetic acid (henceforth, TFA) was added to inactivate the trypsin. Peptides were then desalted and isolated using C18 reverse phase resin ZipTips (Pierce), and subsequently eluted in 50 μL of 50% acetonitrile (ACN)/0.1% TFA (vol/vol). 1 μL of the eluted peptides were spotted in triplicate on a ground steel plate using α-cyano-4-hydroxycinnamic acid as the matrix solution [1% in 50%ACN/0.1% TFA (vol/vol/vol)] in a ratio of 1:1 with eluate. Spots were left to dry for three hours. Mass spectrometry was performed using a Bruker Ultraflex III (Bruker Daltonics) MALDI-TOF-MS instrument in reflector mode with laser acquisition set to 1200. The generated spectral output was analysed using the open-source software mMass v.5.5.0 (www.mmass.org) and peptides were identified based on published unique marker ions [30,31].

## Results

### Radiocarbon dating

The isotopic values and the C:N ratios of all the Fontana Nuova collagen samples fall within acceptable ranges according to standard quality criteria [23]. Moreover, the collagen yields are between 2.1% and 5.4% (Table 1), thus well above the minimum acceptable value (1%). Based on these results, we can confirm the good quality of all the extracts and the validity of all the AMS radiocarbon dates reported in this paper.

**Fig 2:**
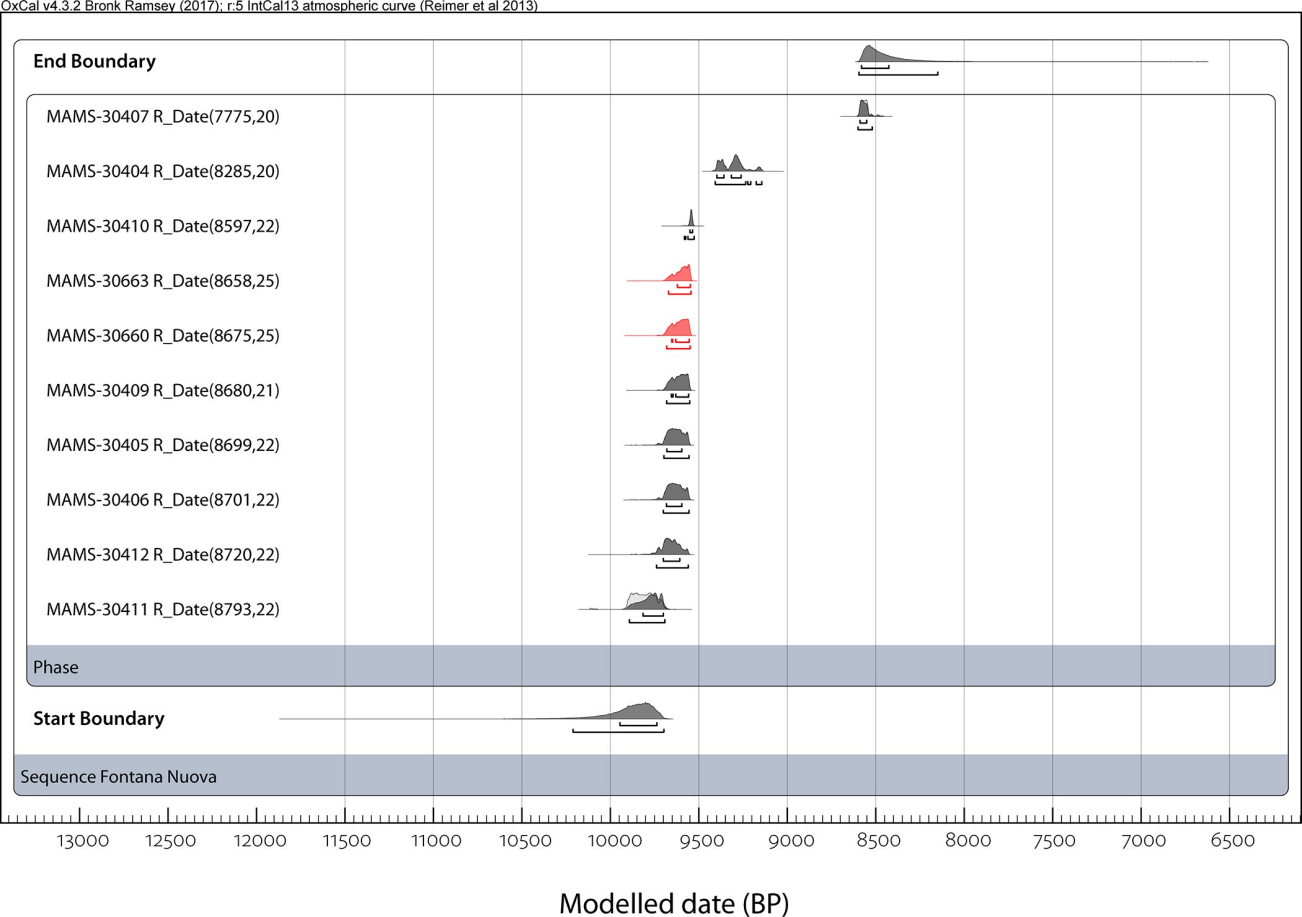
Radiocarbon dates. Plot of the modelled calibrated ages of the ten collagen extracts from human and other faunal skeletal remains recovered at Fontana Nuova. The two human teeth have probability distributions marked in red (MAMS-30660 and MAMS-30663).

We constructed a Bayesian model grouping together all the ^14^C dates as a single phase (Fig 2), given the lack of stratigraphic information. The two humans are in red within the modelled sequences. To build the model, all the ^14^C ages were calibrated with the IntCal13 calibration curve [32] using OxCal v4.3 [33]. Calibrated dates are given in cal BP, with both the 68.2% and 95.4% probability range in Table 2. In this way, we provide estimates for the start and end of Fontana Nuova phase within the model, represented by the different start and end boundaries (Fig 2 and Table 2).

**Table 2:** Modelled radiocarbon dates.

Fontana Nuova	Unmodelled (BP)				Modelled (BP)			
	*from*	*to*	*From*	*to*	*from*	*to*	*from*	*to*
	*68*.*2%*		*95*.*4%*		*68*.*2%*		*95*.*4%*	
**End Boundary**					**8580**	**8430**	**8600**	**8150**
MAMS-30407 (7775,20)	8590	8550	8600	8480	8590	8550	8600	8520
MAMS-30404 (8285,20)	9400	9260	9410	9140	9400	9260	9410	9140
MAMS-30410 (8597,22)	9550	9540	9580	9530	9550	9540	9580	9530
MAMS-30663 (8658,25)	9620	9550	9670	9550	9620	9550	9670	9540
MAMS-30660 (8675,25)	9650	9550	9680	9550	9650	9560	9680	9550
MAMS-30409 (8680,21)	9660	9560	9680	9550	9660	9560	9680	9550
MAMS-30405 (8699,22)	9680	9560	9700	9560	9680	9600	9700	9560
MAMS-30406 (8701,22)	9680	9570	9700	9560	9680	9600	9700	9560
MAMS-30412 (8720,22)	9700	9610	9740	9560	9700	9610	9740	9560
MAMS-30411 (8793,22)	9890	9750	9910	9700	9820	9700	9890	9690
**Start Boundary**					**9950**	**9740**	**10210**	**9700**

Calibrated AMS ^14^C dating of the Fontana Nuova site (unmodelled). Modelled calibrated boundaries and ages of the site performed using OxCal 4.3 [33] with the IntCal13 curve [32].

The 2σ calibrated age ranges are comprised between 9910–9700 cal BP (R-EVA 1880: 8793±22 BP) and 8600–8480 cal BP (R-EVA 1871: 7775±20 BP), which means that they are all early Holocene (and not Late Pleistocene). In addition, it should be noted that, with the exception of two specimens (i.e. R-EVA 1862 and 1871), all other samples have overlapping calibrated age ranges (the overlap is marginal in the case of sample R-EVA 1880, which is the oldest specimen dated in this study). The two human teeth (R-EVA 1895 and 1896) have yielded practically identical dates (respectively 8675±25 BP and 8658±25 BP) and can be considered fully contemporary, which supports the hypothesis proposed by Chilardi et al. [12] (on the basis of their morphology) that these two specimens may belong to the same individual. The start and end boundaries, thus, indicate that Fontana Nuova was surely occupied during the early Holocene, when Sicily was inhabited by Mesolithic, and not Upper Palaeolithic, hunter-gatherers. The human teeth are contemporary with Mesolithic phase II at Grotta dell’Uzzo, whilst the most recent sample (R-EVA 1871) dates to between the late stages of Mesolithic II and the so-called Mesolithic-Neolithic transition [34,35].

### Stable isotope analysis

**Fig 3:**
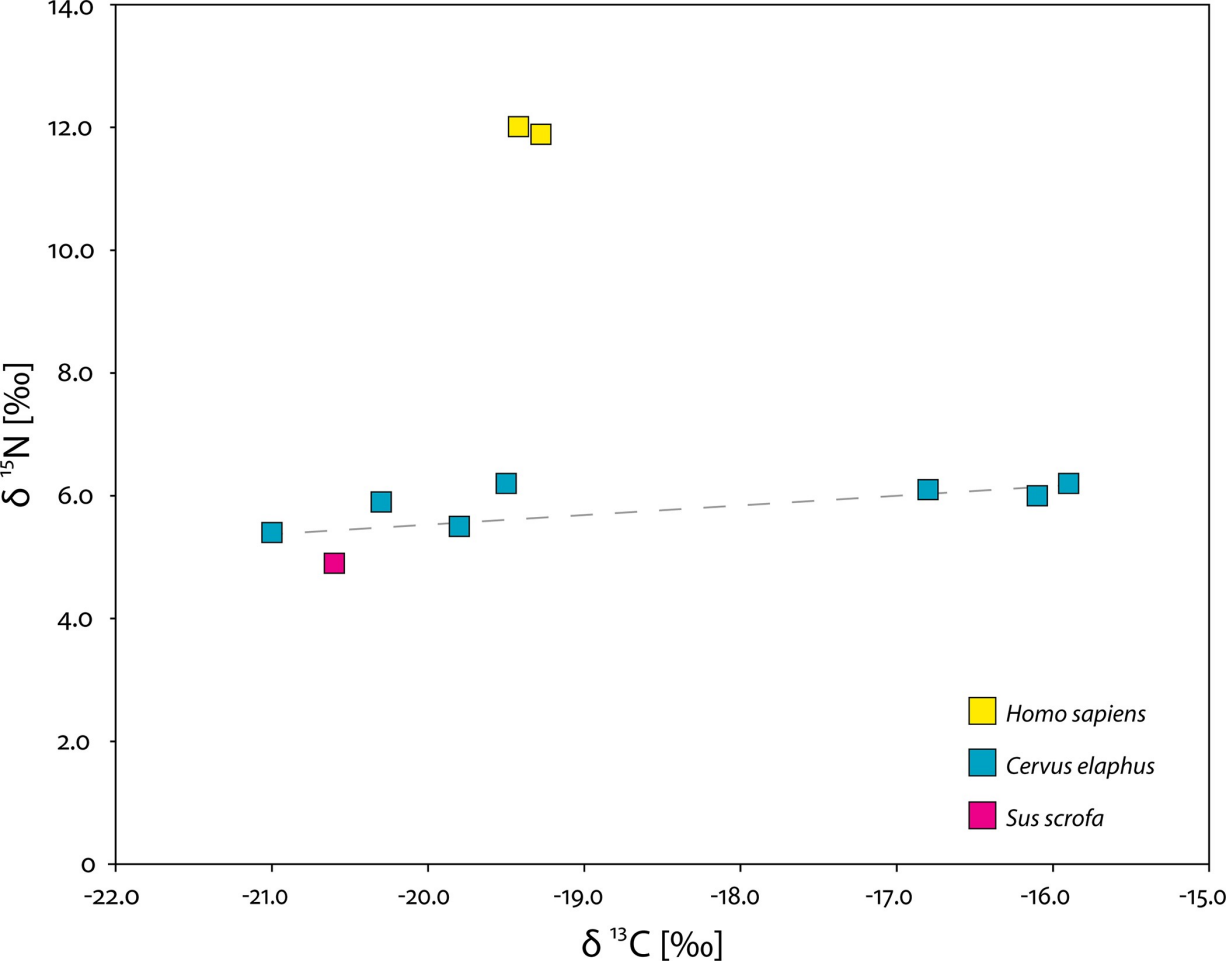
Isotope analyses. Carbon (δ^13^C) and nitrogen (δ^15^N) isotope composition of collagen extracted human and faunal remains recovered at Riparo di Fontana Nuova. The x-axis covers most of the variation in δ^13^C values recorded in Mediterranean contexts from fully terrestrial to marine [36].

The animal bones that yielded well-preserved collagen are 7 *C*. *elaphus* specimens, attributable to at least three individuals, and 1 *S*. *scrofa* specimen (Table 1 and Fig 3). Four red deer and the wild boar specimens have δ^13^C values comprised between -21.0‰ and -19.5‰, which is compatible with the isotopic composition of environments dominated or characterized exclusively by the presence of C_3_ plants. The mean δ^13^C and δ^15^N values for the four red deer alone (given that as discussed above the *S*. *scrofa* is later than the humans analyzed) are respectively -20.2±0.7‰ and 5.8±0.4‰. The δ^15^N of the only *S*. *scrofa* specimen for which data are available has a lower value than red deer, which suggests that the wild boar in question subsisted on plants and did not have a truly omnivorous diet. However, the fact that the single wild boar sample for which we have data is not contemporary to the humans precludes us from attempting to reconstruct the importance of *S*. *scrofa* relative to *C*. *elaphus* in the diet of the occupants of Fontana Nuova.

Three specimens identified as *C*. *elaphus* both on morphological [12] and proteomic grounds, however, have significantly higher δ^13^C values between -16.8‰ and -15.9‰ (mean = -16.3‰ ± 0.5‰). The δ^15^N values of these specimens are all quite similar (range = 0.2‰) and comprised between 6.0‰ and 6.2‰ (mean = 6.1 ± 0.1‰). This evidence suggests that the red deer in question, which as mentioned above may be represented by three or possibly four individuals, fed on a mixed diet of C_3_ and C_4_ plants and that there may have been an extremely large variability in carbon isotope ratios in early Holocene SE Sicily. Red deer are intermediate feeders, given that they are mainly browsers but can obtain around a third of their food from grasses, sedges and forbes [37]. This may explain the isotope composition of specimens R-EVA 1862, 1877 and 1880, although it should be noted that at no other Late Pleistocene or early Holocene Mediterranean site have *C*. *elaphus* individuals with such high δ^13^C values been encountered (e.g. [35,36,38–45]).

This is also not the case of other prehistoric specimens from across Europe, even though the whole range of environments in which red deer can be found have been sampled [46–48]. The high δ^13^C values measured on the bone collagen of R-EVA 1862, 1877 and 1880 are also significantly higher than the range for modern *C*. *elaphus* [49]. In fact, specimens that lived on the Scottish island of Rum had values no higher than -21.2‰, which actually was interpreted as indicating seaweed consumption. Given that the three above-mentioned red deer bones have δ^13^C values averaging -16.3‰, this would be indicative of a consumption of marine foodstuffs that is unknown for these cervids. Moreover, had the red deer specimens with enriched ^13^C consumed seagrasses or seaweed we would expect them to have had different δ^15^N values relative to their conspecifics, which is not the case. Other possible plants that may have been consumed by the *C*. *elaphus* in question are sedges and forbes or grasses (including weeds such as *Cynodon dactylon*) [45] with C_4_ photosynthetic pathway, neither of which have been attested for prehistoric wild mammals on the northern side of the Mediterranean basin. Similarly, high δ^13^C values have been recorded in modern *C*. *elaphus* from North America, albeit from metabolically more active tissues than bone collagen (i.e. muscle) and not even in tissues with more intermediate turnover times such as the hoof [50,51]. These carbon isotope compositions are attained in C_4_ grasslands, but even in such cases deer prefer feeding on C_3_ plants [50], with possible increases in C_4_ plant consumption during warm periods of the year [51]. Further research is needed to establish whether south-eastern Sicily and southern Europe were characterized by the presence of C_4_ grasslands and, if so, how *C*. *elaphus* may have adapted to such conditions. Another theoretically possible interpretation of the high δ^13^C values is that the red deer in question were not local and had been introduced from parts of the Mediterranean Basin where C_4_ plants were widely present even in prehistoric times, although it is not clear why this should have been done given the abundance of this ungulate in Sicily during the early Holocene.

The dentinal collagen extracted from the two human teeth has very similar isotopic compositions (Table 1; Fig 2), suggesting that they may belong to the same individual, as hypothesized by Chilardi et al. [12]. The δ^13^C values are enriched by around 1.0‰ relative to the mean (= 20.2 ± 0.6‰) for the faunal specimens typical of environments dominated by C_3_ plants. The δ^15^N values are around 6.2‰ higher than the mean for the four deer specimens contemporary to the humans and typical of environments dominated by C_3_ plants. These offsets suggest that the meat of terrestrial animals, such as *C*. *elaphus* (and possibly *S*. *scrofa*), were the main sources of dietary protein.

However, given that especially the nitrogen isotope values are offset by more than what is generally thought to be the case for a consumer-prey relationship (e.g. [52]), it is likely that animal resources other than those for which isotope values are available may have contributed around a tenth or more to the diet. One possible species that may have been consumed by the hunter-gatherers of Fontana Nuova is *Bos primigenius*, which in the Mediterranean context is known to have had higher δ^15^N values than *C*. *elaphus* (e.g. [47]). Other possible foods that may result in higher δ^15^N values are aquatic resources, such as freshwater and/or marine fauna. As in the case of the Mesolithic hunter-gatherers of Grotta dell’Uzzo [35], however, these are unlikely to have represented important foodstuffs in absolute terms. Overall, the isotopic composition of the two human teeth is typical of Late Pleistocene and early Holocene Mediterranean hunter-gatherers who relied heavily on terrestrial animal protein and partly on aquatic foods (e.g. [35,36,40,41,53]).

### aDNA

4.5 and 5.8 million raw reads were generated respectively for samples R-EVA 1895 and R-EVA 1896. We could not identify any authentic human sequences for sample R-EVA 1895. Six reads could be aligned to the human mitochondrial genome and 429 reads to the nuclear genome of sample R-EVA 1896. The low content of authentic human DNA in sample R-EVA 1986 did not allow any further analysis.

### ZooMS

Peptide mass fingerprinting by ZooMS followed established protocols. Seven (i.e. R-EVA 1862, 1866, 1871, 1877, 1878, 1880, 1881) samples produced spectra that enabled species identification that were in agreement with morphological examination as part of traditional zooarchaeological analysis (see Table 3). One sample (R-EVA 1865) did not contain preserved peptides to allow for species identification. Six samples were identified as red deer and one as wild boar (spectral triplicate raw data has been uploaded to ADS: https://doi.org/10.5284/1049180). ZooMS is not currently able to distinguish between red deer (*Cervus elaphus*) and European elk (*Alces alces*). This is because the amino acid sequences of their collagen triple helices are almost indistinguishable, and the few peptides displaying differences do not ionize under the current experimental conditions of the MALDI-TOF-MS. However, since elk was not part of the endemic fauna of Sicily during this period [54], the bones in question can be attributed to red deer with confidence.

**Table 3:** ZooMs analyses.

Sample name	Laboratory number (R-EVA)	Visual ID	Element	weight mg	P1	A1	A2	B	C	P2	D	E	F1	F2	G1	G2	Barcode ID
Fon-02	1862	*Cervus elaphus*	humerus	16.2	1105.5	trace	1196.6	1427.7	1550.7	1648.8	2131.1	trace	2883.3	2899.3	3018.*	3034.3*	*Cervus elaphus*
Fon-05	1865	*Cervus elaphus*	scafocuboid	12.2	-	-	-	-	-	-	-	-	-	-	-	-	NoID
Fon-06	1866	*Cervus elaphus*	radius	11.1	1105.7	1180.6	1196.6	1427.7	1550.7	1648.8	2131.1	trace	2883.3	2899.3	3018.2*	3034.2*	*Cervus elaphus*
Fon-11	1871	*Sus scrofa*	Metacarpal III	23.5	1105.5	-	trace	1453.8	1550.8	1648.8	2131.1	-	-	-	-	-	*Sus scrofa*
Fon-17	1877	*Cervus elaphus*	tibia	17.1	1105.5	1180.5	1196.5	1427.7	1550.7	1648.8	2131.1	-	2883.3	trace	3019.3*	-	*Cervus elaphus*
Fon-18	1878	*Cervus elaphus*	tibia	13.2	1105.5	1180.6	1196.6	1427.7	1550.8	1648.8	2131.1	-	2883.3	2899.3	3019.3*	3034.3*	*Cervus elaphus*
Fon-20	1880	*Cervus elaphus*	tibia	24.7	1105.5	-	1196.5	1427.7	1550.7	1648.8	2131.1	-	2883.2	-	-	-	*Cervus elaphus*
Fon-21	1881	*Cervus elaphus*	phalanx I	10.8	1105.5	1180.6	1196.6	1427.7	1550.7	1648.8	2131.1	trace*	2883.3	trace	3019.2*	3034.4*	*Cervus elaphus*

Unique species identification peptides observed by MALDI-TOF-MS.

*Deamidated. 1 Da mass shift.

## Discussion

The main outcome of our study is to have clarified the chronology of Fontana Nuova, demonstrating that the faunal and human remains, used by Chilardi and colleagues to back up their attribution of the site to the Aurignacian [12], actually date to the Holocene (9900–8500 cal. BP), when Sicily was occupied by Mesolithic hunter-gatherers. An attribution of the lithic assemblage to the Aurignacian can now be rejected, not only due to the results of our radiocarbon dating, but also given that the lithic assemblage from this site has been re-assigned to the Late Epigravettian by Lo Vetro and Martini [13]. In fact, the finds only include 2 strangulated blades (both not intact, which makes their typological attribution more speculative) and 6 Aurignacian blades [7], representing a small fraction of the overall complex (ca. 6%), and do not include any animal bone tools, typical of the early Upper Palaeolithic culture in question. Given the similarity between Late Epigravettian and Mesolithic (‘Undifferentiated Epigravetian’) industries in Sicily (where some of the techno-typological features of the latter are rooted in the Epigravettian tradition) [13,55] and in the absence of a new study of the lithic assemblage, it is not possible to exclude that the site of Riparo di Fontana Nuova, as many others on the island, was occupied by hunter-gatherers of both cultures. However, this hypothetical possibility is not the most parsimonious interpretation of the radiocarbon data available for the site, because it would imply that: (1) its small and seemingly discrete assemblage is mixed, and that (2) collagen is preserved by chance only on bones of Holocene/Mesolithic age, whilst the remains that did not yield collagen are Late Pleistocene/Palaeolithic, or that (3) the lithics are mixed, but the fauna is not. Based on our experience on other prehistoric sites on Sicily, the kind of differential preservation of collagen necessary to support these hypotheses has never been recorded at sites occupied in both periods. Moreover, these hypothetical explanations contrast both with Bernabò Brea’s on-site observation that all the material originated from the discrete middle layer [14] and with the nature of the material culture and skeletal assemblages suggesting that Fontana Nuova was occupied infrequently and short-term [12]. The fact that all but two of the calibrated age ranges for the specimens dated in this study overlap at 2σ and that the overall modelled age range covers only a period of between 2060 and 1100 calibrated years is fully compatible with an infrequently occupied short-term site.

Overall, there are no more grounds to support the interpretation that Riparo di Fontana Nuova was the southernmost Aurignacian site in Europe [12], as well as the notion arising from it that early Upper Palaeolithic hunter-gatherers crossed the Strait of Messina [2,56]. Paradigm-shifting attributions such as the one made for Fontana Nuova should be based on solid evidence and chronological frameworks, which is not the case in point, especially considering that there are no other Aurignacian sites on Sicily or on any other Mediterranean island. The oldest absolutely-dated archaeological sites in Sicily are, thus, Riparo del Castello (OxA-10040 13485 ±80, 16,900–15,600 cal. BP 2σ) [57] and Grotta delle Uccerie (LTL1517A 13191 ±120) [58], given that the earliest date for Acqua Fitusa has been questioned [59]. The oldest directly-dated human remains, on the other hand, are from Grotta Addaura Caprara (KIA-36055 12890 ±60 BP: 15,950–15,007 cal. BP 2σ) and Grotta di San Teodoro (ETH-34451 12580 ±130 BP: 15,232–14,126 cal. BP 2σ) [40]. This implies that the only undisputed evidence for an Upper Palaeolithic occupation of Sicily is represented by post-LGM, Epigravettian sites and even the plausible hypothesis that the peopling of Sicily may have occurred at the time of the emergence of the land bridge between the island and Calabria (around 21.5–20.0 ka cal. BP) [16] requires evidence in the form of archaeological sites earlier than Riparo del Castello and Grotta delle Uccerie.

A second outcome of our study is to have generated a small isotope dataset that allows us to attempt a broad reconstruction of Mesolithic diets in south-eastern Sicily. The carbon isotope composition of the two analysed human teeth is similar to that of other Mesolithic individuals from Sicily [35,36,41], demonstrating that Holocene hunter-gatherers on the island had a subsistence that was heavily based on hunting and on the consumption of the meat of terrestrial mammals. The only difference between the isotope composition of the teeth from Fontana Nuova and of other Mesolithic human remains from Sicily is represented by their high nitrogen values, which may indicate some reliance on aquatic foods or a different nitrogen isotope baseline in this part of the island.

The possibility of different isotopic baselines should also be considered in attempts to explain the high carbon isotope values on three *C*. *elaphus* specimens, for instance in connection to short-term climatic and environmental instability attested in Sicily during the early Holocene [60]. This hypothesis can probably be discounted, given that one of the deer samples (R-EVA 1862) does not overlap chronologically with the other two and that a similar hypothesis would thus imply an anomalous niche partitioning by red deer. In fact, the high carbon isotope ratios of R-EVA 1862, 1877 and 1880 may be indicative either of consumption of C_4_ plants from extensive grasslands (the presence of which is not attested in south-eastern Sicily), of forbes, sedges and wetland C_4_ vegetation (although it is not clear where such environments would have been located in the case of Fontana Nuova) or of their non-local origin. In the absence of human intervention in deer feeding, the first two scenarios would, as mentioned above, imply an unprecedented degree of niche partitioning within the same species, which is not likely in nature. In the latter case, it is possible that the deer originated from regions with a relatively high proportion of C_4_ grasses, which are more common around the eastern or southern coasts of the Mediterranean Sea, as hypothesized for an ovicaprid with similar isotopic values (δ^13^C: -16.2‰; δ^15^N: 6.3‰) from the site of Grotta d’Oriente on Favignana in western Sicily [41]. Another working hypothesis is that the three bones in question belonged to individuals that had been fed by humans, as part of proto-breeding of *C*. *elaphus*, which is something that has been speculatively claimed only for protohistoric times in Sicily [61]. This has also been hypothesized in the case of cervids in Cyprus [62], where it has been demonstrated that ungulates were introduced by boat in efforts by foragers to maintain viable stocks for hunting [63]. However, given that *C*. *elaphus* was common in Sicily during the early Holocene, it is not clear why these animals should have been introduced. To date the only prehistoric ungulates from southern European contexts that have been found to have δ^13^C values as high as the three deer specimens from Fontana Nuova are domestic cattle specimens from the Late Neolithic site of Makriyalos in northern Greece [45]. Recent analyses on *Bos taurus* from this site have shown that some individuals acquired large proportions of their feed from pastures rich in C_4_ vegetation that may have included weeds such as *Cynodon dactylon* or, more likely, plants in marshy coastal environments enriched in ^13^C. In the case of the cattle from Makriyalos, their feeding areas were influenced by human management, whilst it is not clear why, if the ^13^C-enriched *C*. *elaphus* were actually feeding in coastal marshes, this behavior has not been documented in any of their conspecifics elsewhere in the Mediterranean or further afield.

## Conclusions

The AMS radiocarbon dates on collagen from ten skeletal remains retrieved at Riparo di Fontana Nuova assign both the fauna and humans to the Holocene. As these were used by Chilardi et al. [12] to argue an early Upper Palaeolithic occupation, we believe that the attribution of the site to the Aurignacian should be discarded. A late Upper Palaeolithic (i.e. Late Epigravettian) occupation cannot be discounted outright, although hypothesizing it requires a non-parsimonious explanation: either that the lithic assemblage is mixed but the bones are not or that by chance collagen was only preserved on bones of Holocene age. There are, thus, no longer credible claims for early Upper Palaeolithic sea crossings to large Mediterranean islands, which has important implications for our knowledge of prehistoric voyaging in this enclosed sea. As far as Sicily is concerned, our findings hopefully signal the end of speculation on the peopling of the island based on materials recovered from undocumented contexts and poorly-dated sites. Only new surveying and excavation campaigns will, thus, enable us to explore further when the largest Mediterranean island was first settled by anatomically modern humans.

## Supporting information

S1 Table Human and faunal skeletal remains sampled for isotopic and radiocarbon analyses. (DOCX)

S1 Fig Three-dimensional digital model of the right maxillary M2. (JPG)

S2 Fig Three-dimensional digital model of the left maxillary P3. (JPG)

S3 Fig *Cervus elaphus*, humerus (Fon-1), before the sampling. (JPG)

S4 Fig *Cervus elaphus*, humerus (Fon-2), before the sampling. (JPG)

S5 Fig *Cervus elaphus*, humerus (Fon-3), before the sampling. (JPG)

S6 Fig *Cervus elaphus*, fermur (Fon-4), before the sampling. (JPG)

S7 Fig *Cervus elaphus*, scaphucuboid (Fon-5), before the sampling. (JPG)

S8 Fig *Cervus elaphus*, radius (Fon-6), before the sampling. (JPG)

S9 Fig *Cervus elaphus*, ulna (Fon-7), before the sampling. (JPG)

S10 Fig *Cervus elaphus*, humerus (Fon-8), before the sampling. (JPG)

S11 Fig *Sus scrofa*, metatarsal II (Fon-9), before the sampling. (JPG)

S12 Fig *Sus scrofa*, metacarpal II (Fon-10), before the sampling. (JPG)

S13 Fig *Sus scrofa*, metacarpal III (Fon-11), before the sampling. (JPG)

S14 Fig *Bos primigenius*, cuneiform (Fon-12), before the sampling. (JPG)

S15 Fig *Bos primigenius*, vertebra (Fon-13), before the sampling. (JPG)

S16 Fig *Bos primigenius*, molar (Fon-14), before the sampling. (JPG)

S17 Fig *Cervus elaphus*, molar III (Fon-15), before the sampling. (JPG)

S18 Fig *Cervus elaphus*, molar I or II (Fon-16), before the sampling. (JPG)

S19 Fig *Cervus elaphus*, tibia, right (Fon-17), before the sampling. (JPG)

S20 Fig *Cervus elaphus*, tibia, right (Fon-18), before the sampling. (JPG)

S21 Fig *Cervus elaphus*, tibia, right (Fon-19), before the sampling. (JPG)

S22 Fig *Cervus elaphus*, tibia, right (Fon-20), before the sampling. (JPG)

S23 Fig *Cervus elaphus*, phalanx I (Fon-21), after the sampling. (JPG)

S24 Fig *Cervus elaphus*, calcaneus (Fon-22), after the sampling. (JPG)

S25 Fig *Homo sapiens*, parietal fragment (Fon-23), before the sampling. (JPG)

S26 Fig *Homo sapiens*, molar II (Fon-24), before the sampling. (JPG)

S27 Fig *Homo sapiens*, premolar III (Fon-25), before the sampling. (JPG)

S1 Appendix OxCal code. (DOCX)
