# Supplementary material for: Radiocarbon dating and isotope analysis on the purported Aurignacian skeletal remains from Fontana Nuova (Ragusa, Italy)
Source: PLoS One. 2019 Mar 20;14(3):e0213173. doi: 10.1371/journal.pone.0213173 (PMC6426221; doi:10.1371/journal.pone.0213173)
Supplement: S1 Table — (DOCX) [file pone.0213173.s001.docx]

**Table S1. Human and faunal skeletal remains sampled for isotopic and radiocarbon analyses**

| Submitter number | R-EVA number | Species | Element |
| --- | --- | --- | --- |
| FON-01 | 1861 | *Cervus elaphus* | humerus |
| FON-02 | 1862 | *Cervus elaphus* | humerus |
| FON-03 | 1863 | *Cervus elaphus* | humerus |
| FON-04 | 1864 | *Cervus elaphus* | femur |
| FON-05 | 1865 | *Cervus elaphus* | scaphocuboid |
| FON-06 | 1866 | *Cervus elaphus* | radius |
| FON-07 | 1867 | *Cervus elaphus* | ulna |
| FON-08 | 1868 | *Cervus elaphus* | humerus |
| FON-09 | 1869 | *Sus scrofa* | metatarsal II |
| FON-10 | 1870 | *Sus scrofa* | metacarpal II |
| FON-11 | 1871 | *Sus scrofa* | metacarpal III |
| FON-12 | 1872 | *Bos primigenius* | cuneiform |
| FON-13 | 1873 | *Bos primigenius* | vertebra (10213) |
| FON-14 | 1874 | *Bos primigenius* | molar (10214) |
| FON-15 | 1875 | *Cervus elaphus* | molar III |
| FON-16 | 1876 | *Cervus elaphus* | molar I or II |
| FON-17 | 1877 | *Cervus elaphus* | tibia (right) |
| FON-18 | 1878 | *Cervus elaphus* | tibia (right) |
| FON-19 | 1879 | *Cervus elaphus* | tibia (right) |
| FON-20 | 1880 | *Cervus elaphus* | tibia (right) |
| FON-21 | 1881 | *Cervus elaphus* | phalanx I |
| FON-22 | 1882 | *Cervus elaphus* | calcaneus |
| FON-23 | 1883 | *Homo sapiens* | parietal fragment |
| FON-24 | 1895 | *Homo sapiens* | molar II |
| FON-25 | 1896 | *Homo sapiens* | premolar III |
