# Supplementary figures and images for: Radiocarbon dating and isotope analysis on the purported Aurignacian skeletal remains from Fontana Nuova (Ragusa, Italy)
Source: PLoS One. 2019 Mar 20;14(3):e0213173. doi: 10.1371/journal.pone.0213173 (PMC6426221; doi:10.1371/journal.pone.0213173)

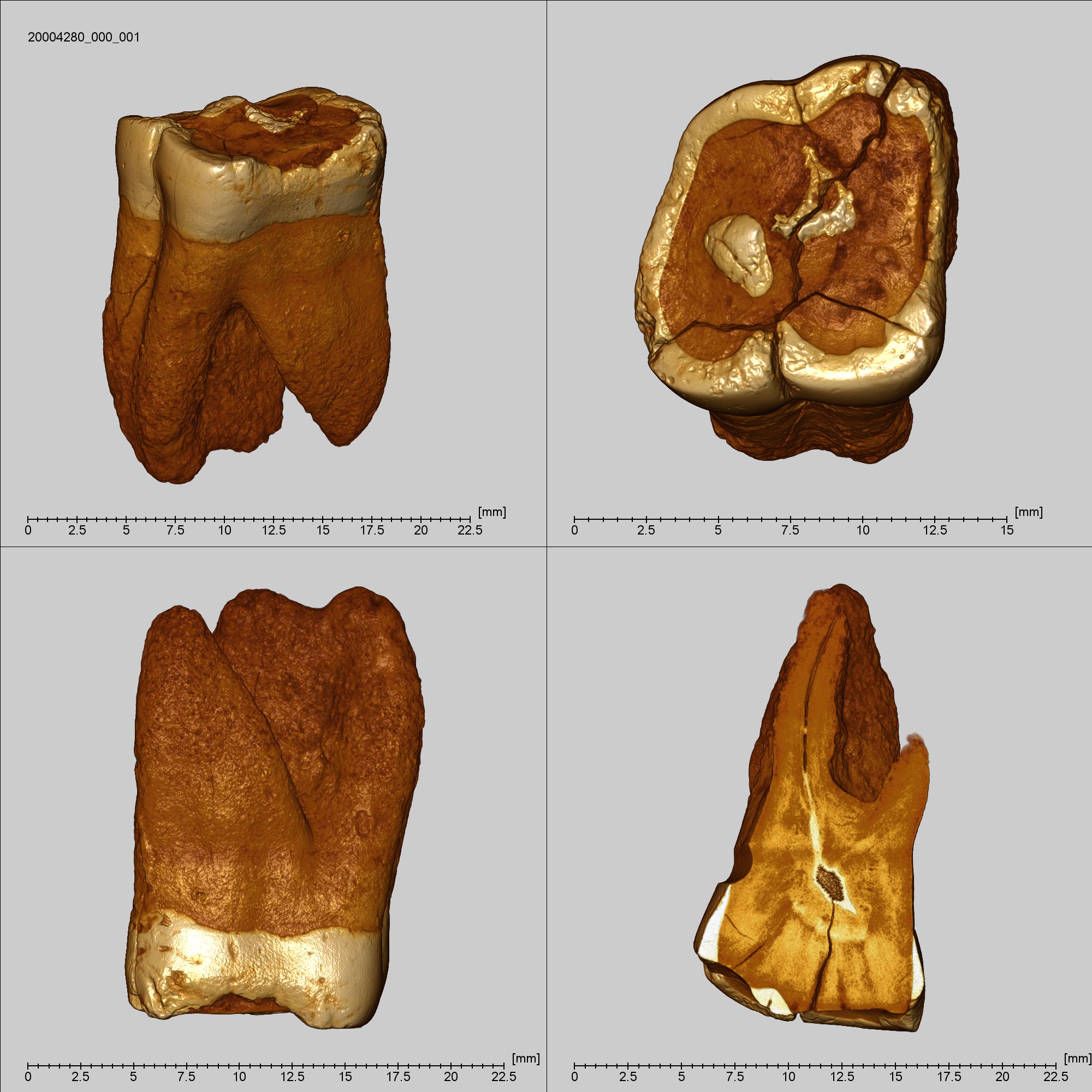

Supplement: S1 Fig — (JPG) [file pone.0213173.s002.jpg]

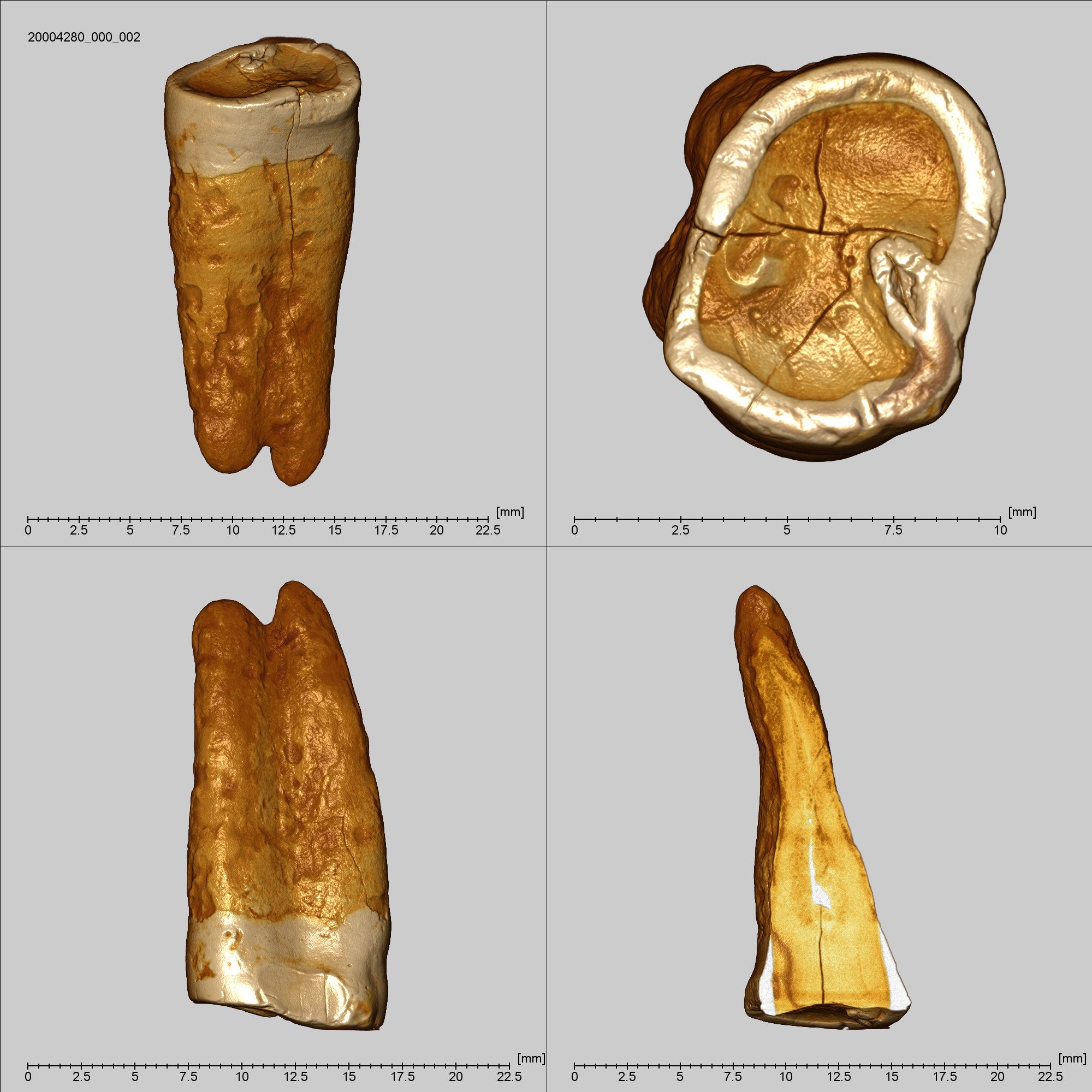

Supplement: S2 Fig — (JPG) [file pone.0213173.s003.jpg]

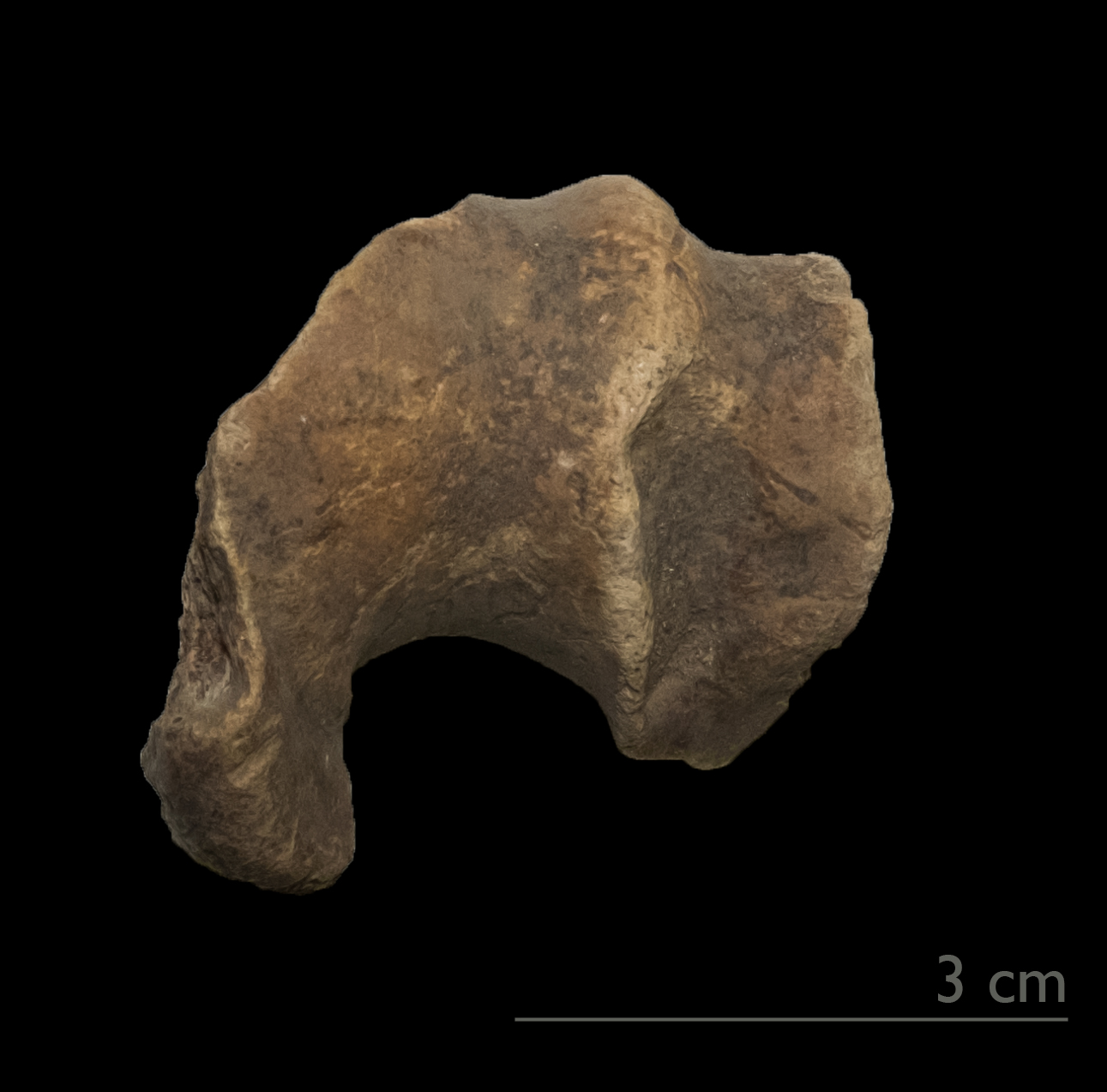

Supplement: S3 Fig — (JPG) [file pone.0213173.s004.jpg]

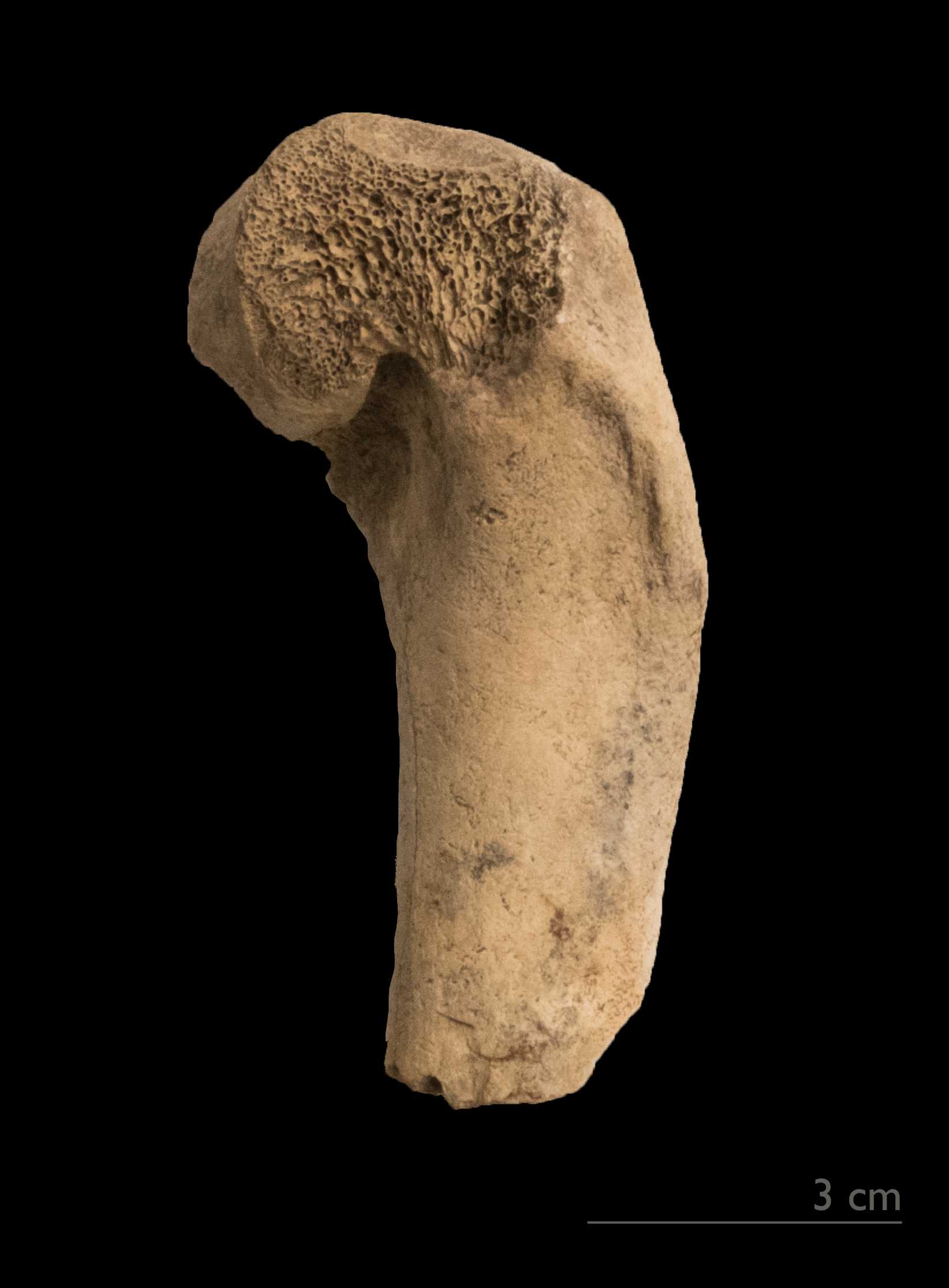

Supplement: S4 Fig — (JPG) [file pone.0213173.s005.jpg]

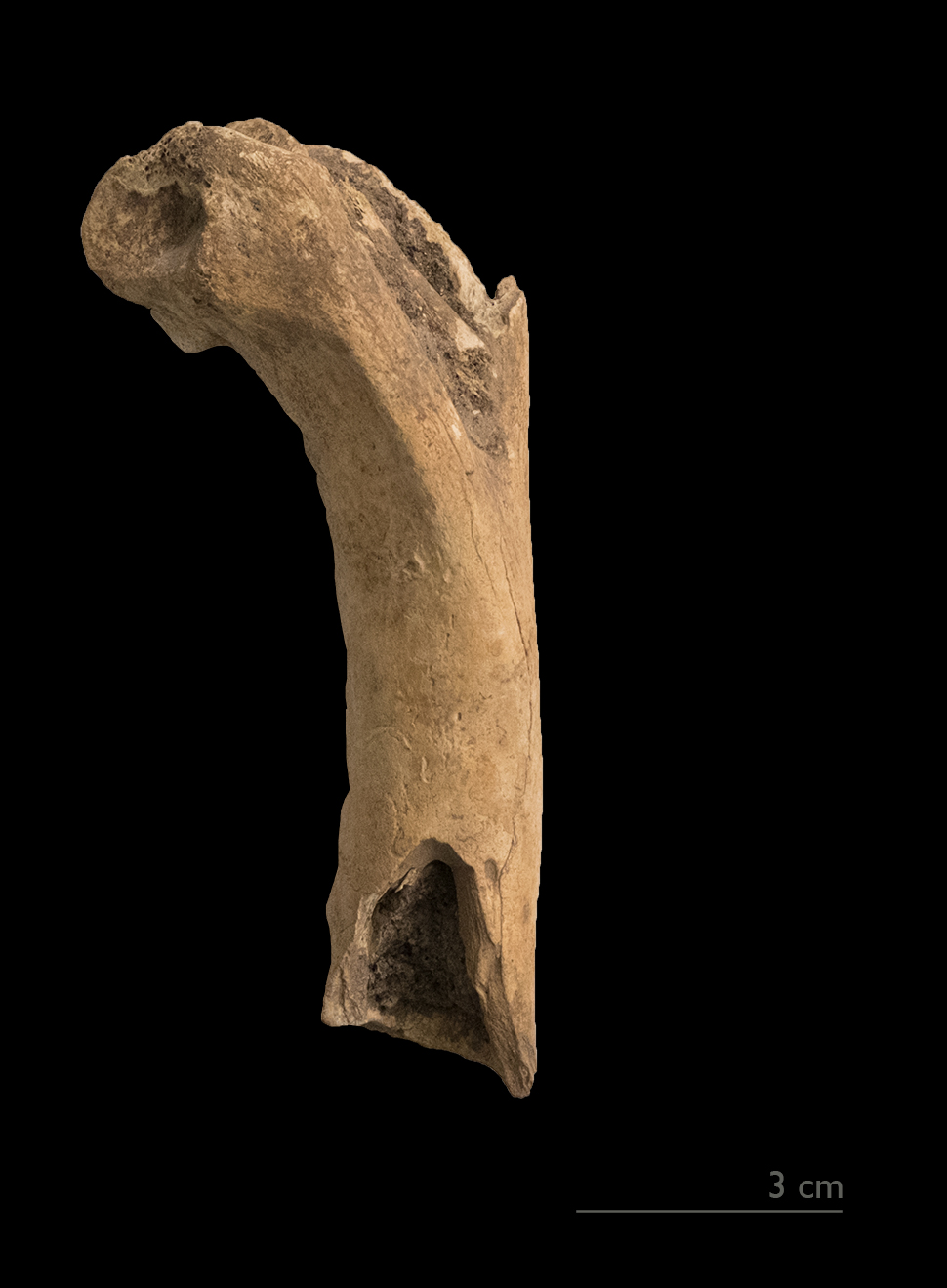

Supplement: S5 Fig — (JPG) [file pone.0213173.s006.jpg]

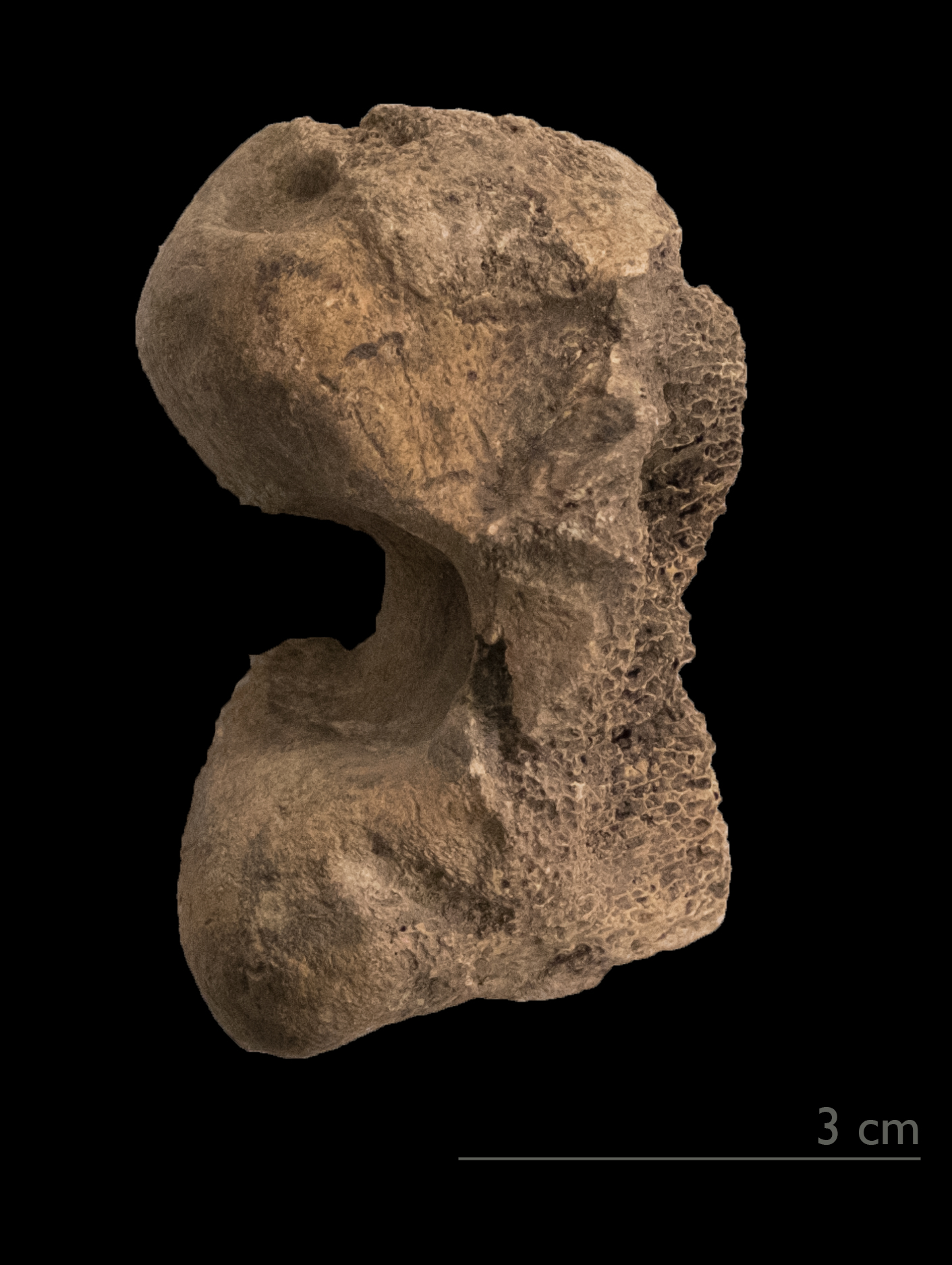

Supplement: S6 Fig — (JPG) [file pone.0213173.s007.jpg]

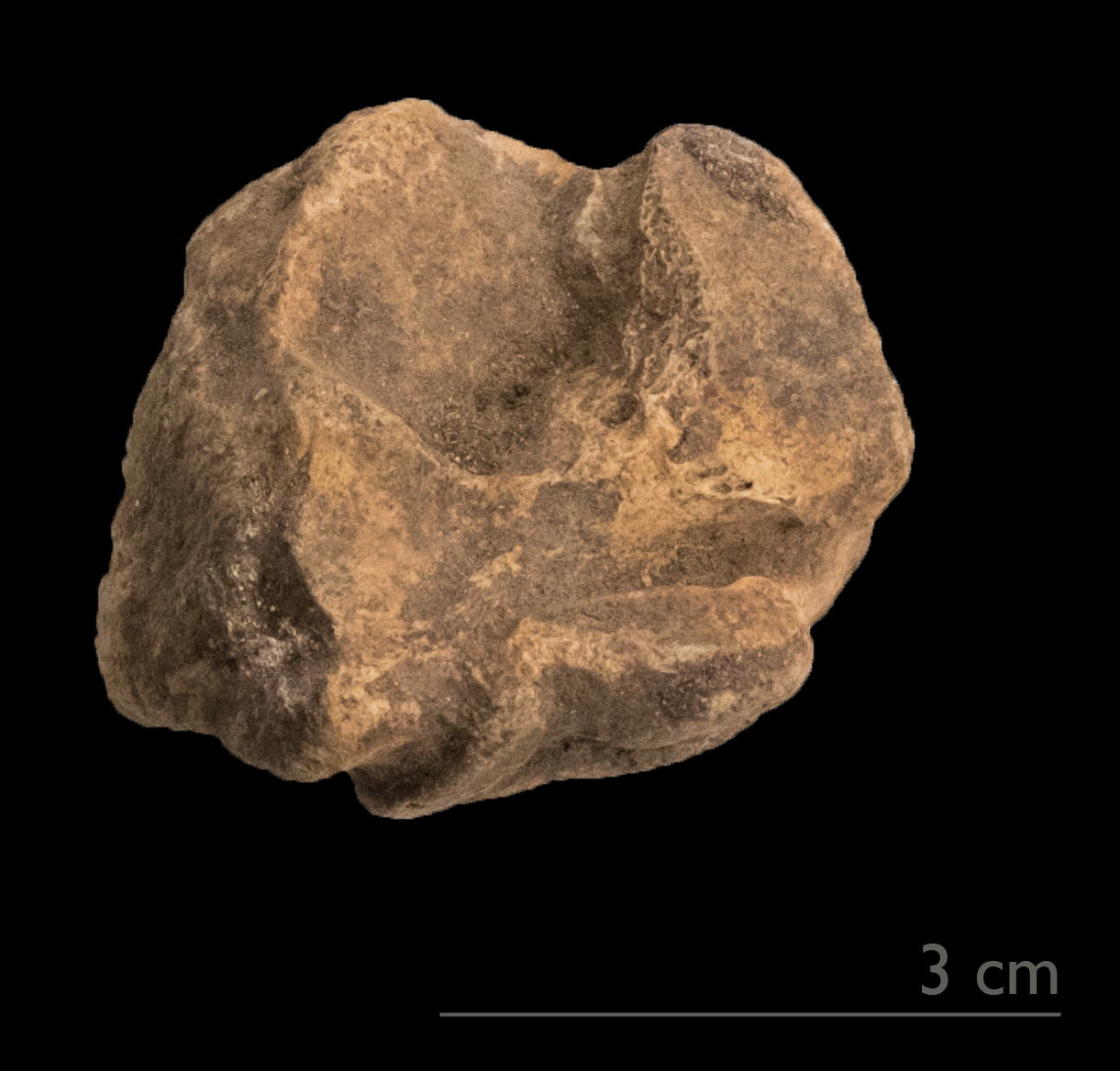

Supplement: S7 Fig — (JPG) [file pone.0213173.s008.jpg]

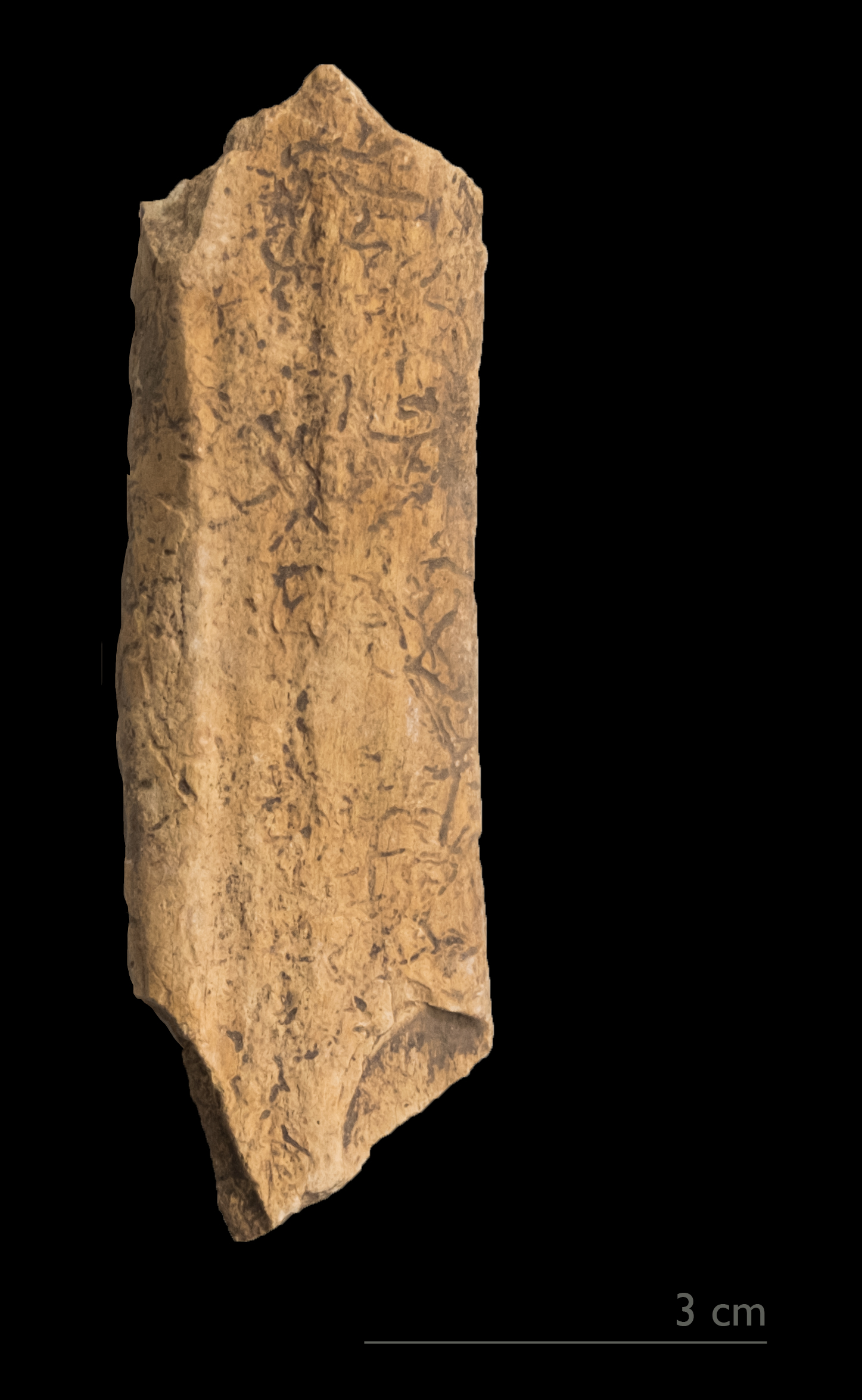

Supplement: S8 Fig — (JPG) [file pone.0213173.s009.jpg]

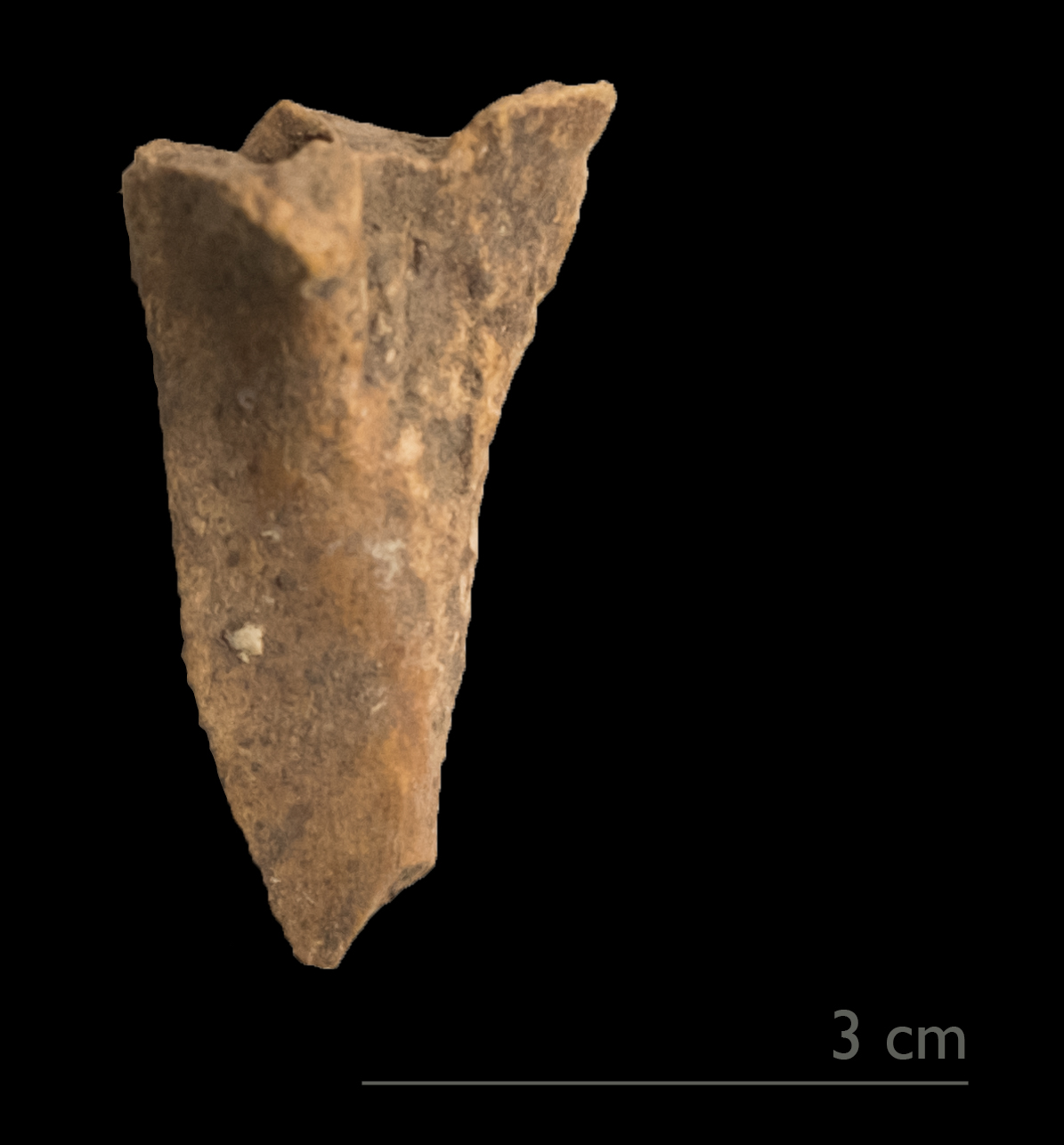

Supplement: S9 Fig — (JPG) [file pone.0213173.s010.jpg]

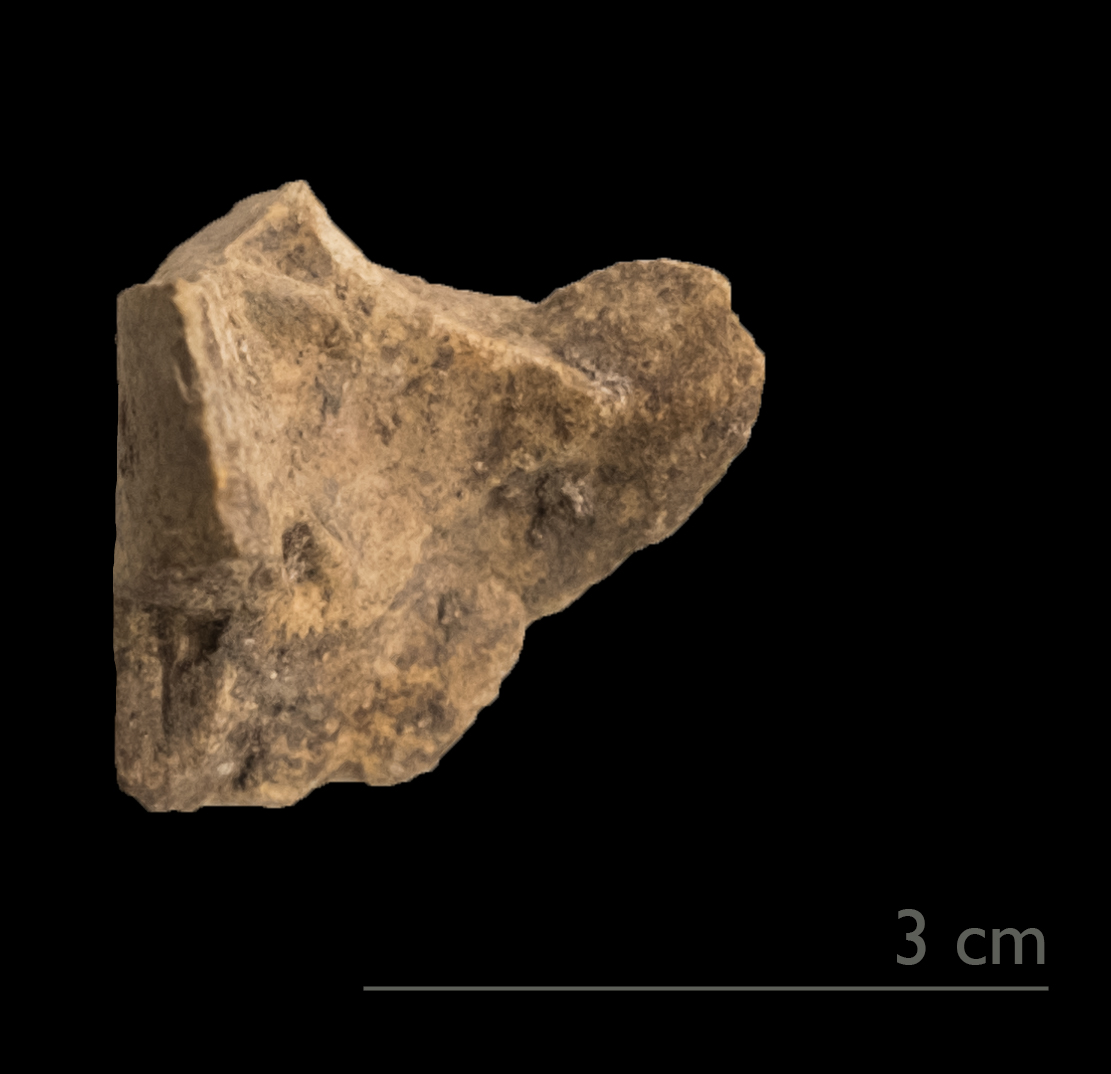

Supplement: S10 Fig — (JPG) [file pone.0213173.s011.jpg]

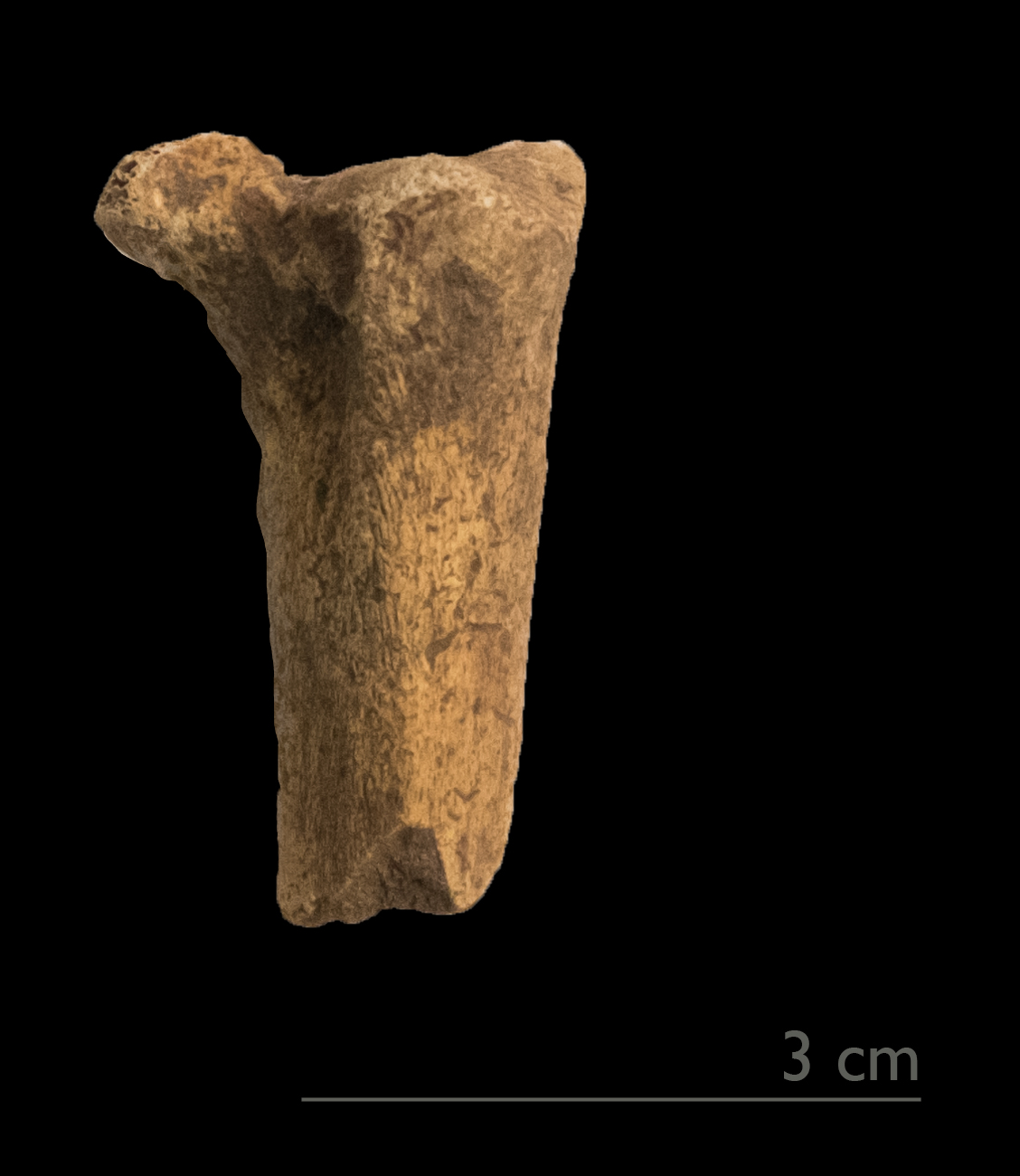

Supplement: S11 Fig — (JPG) [file pone.0213173.s012.jpg]

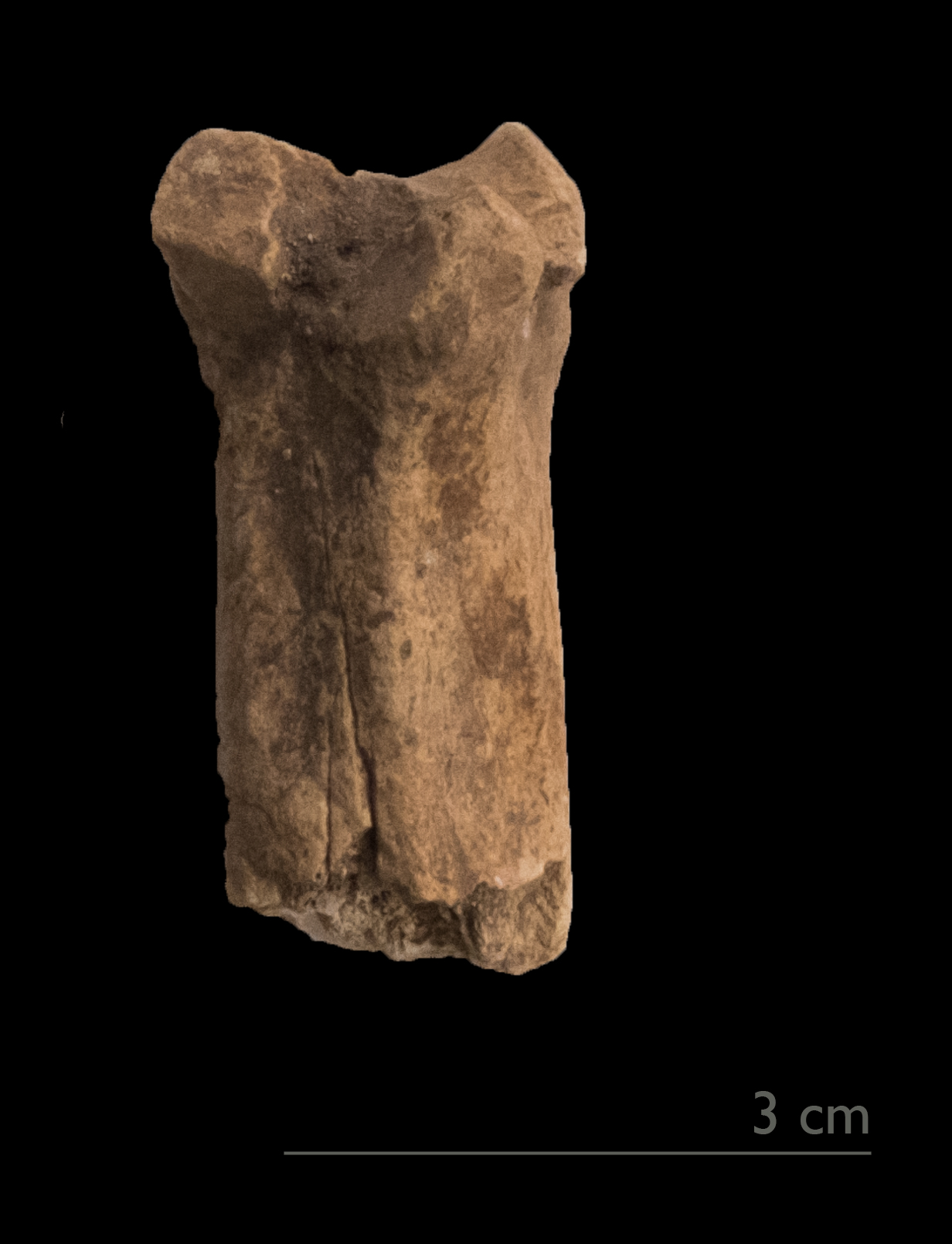

Supplement: S12 Fig — (JPG) [file pone.0213173.s013.jpg]

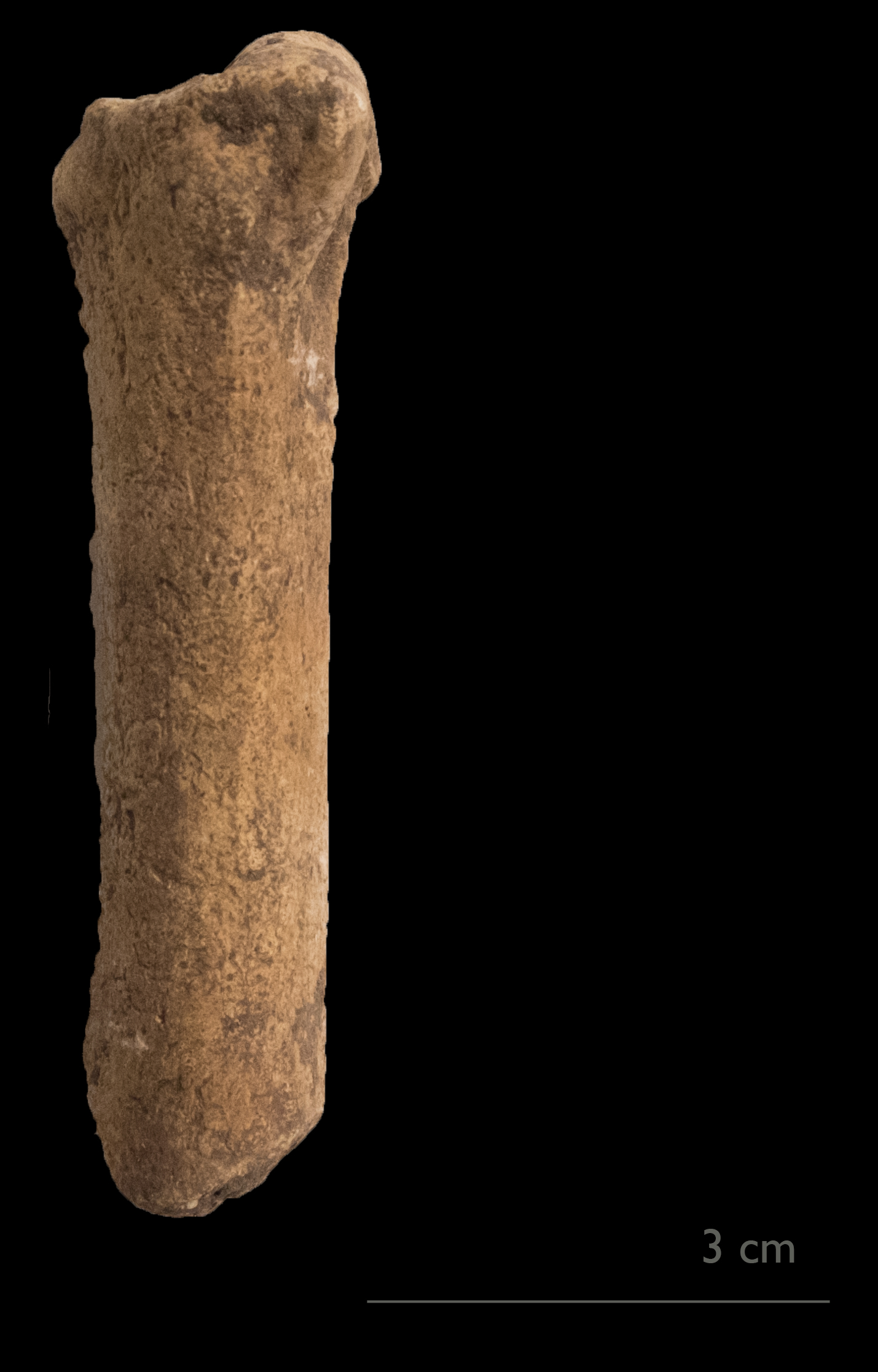

Supplement: S13 Fig — (JPG) [file pone.0213173.s014.jpg]

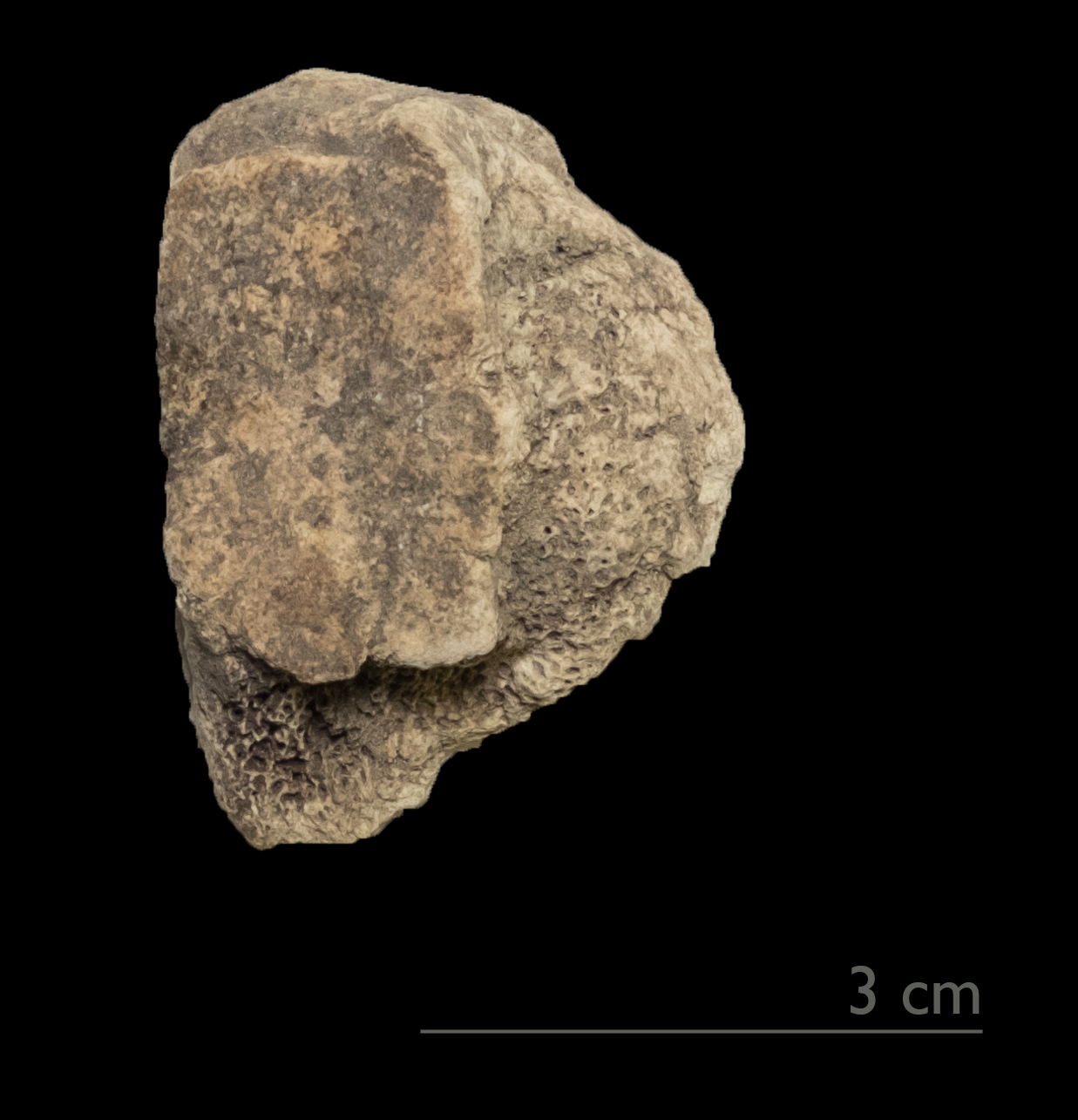

Supplement: S14 Fig — (JPG) [file pone.0213173.s015.jpg]

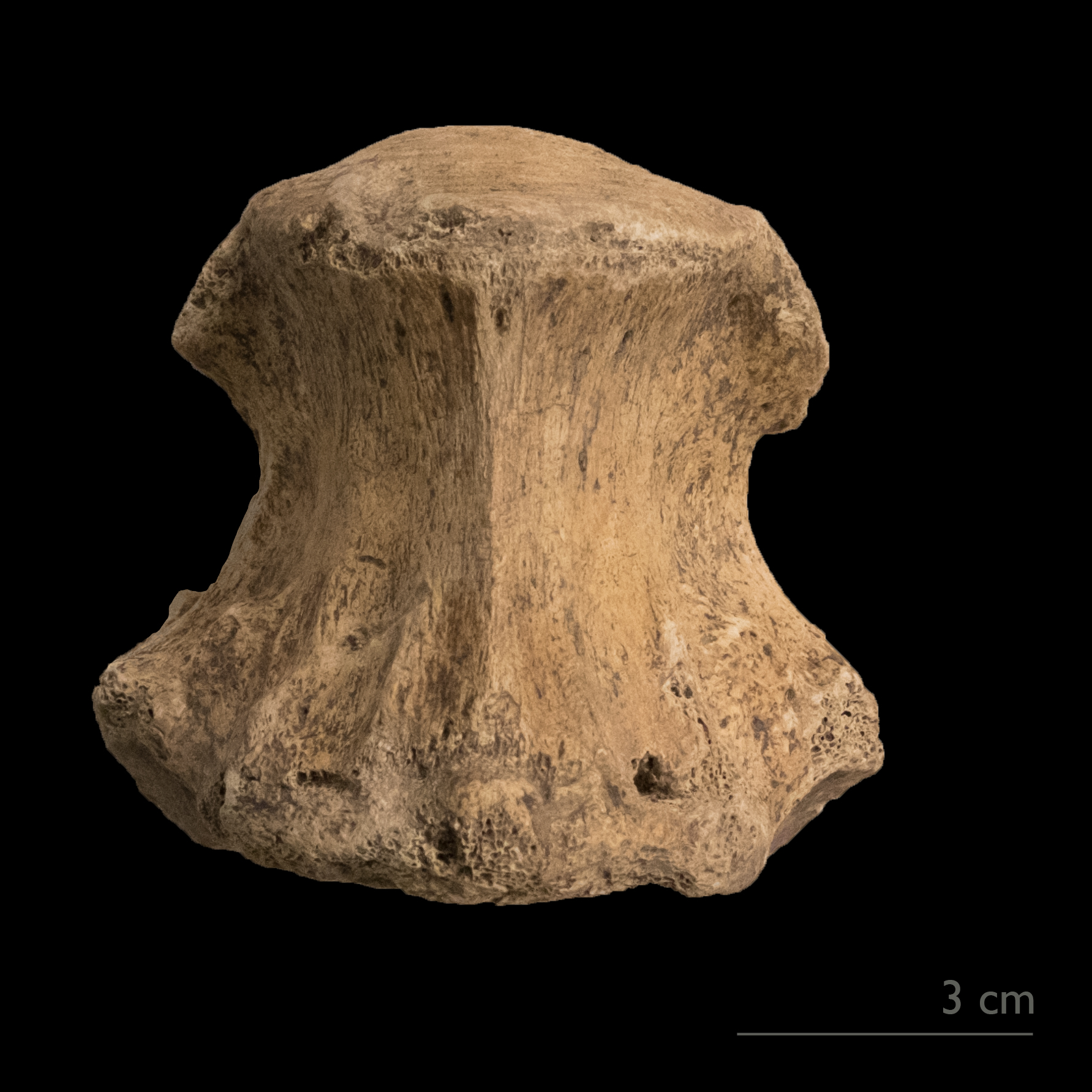

Supplement: S15 Fig — (JPG) [file pone.0213173.s016.jpg]

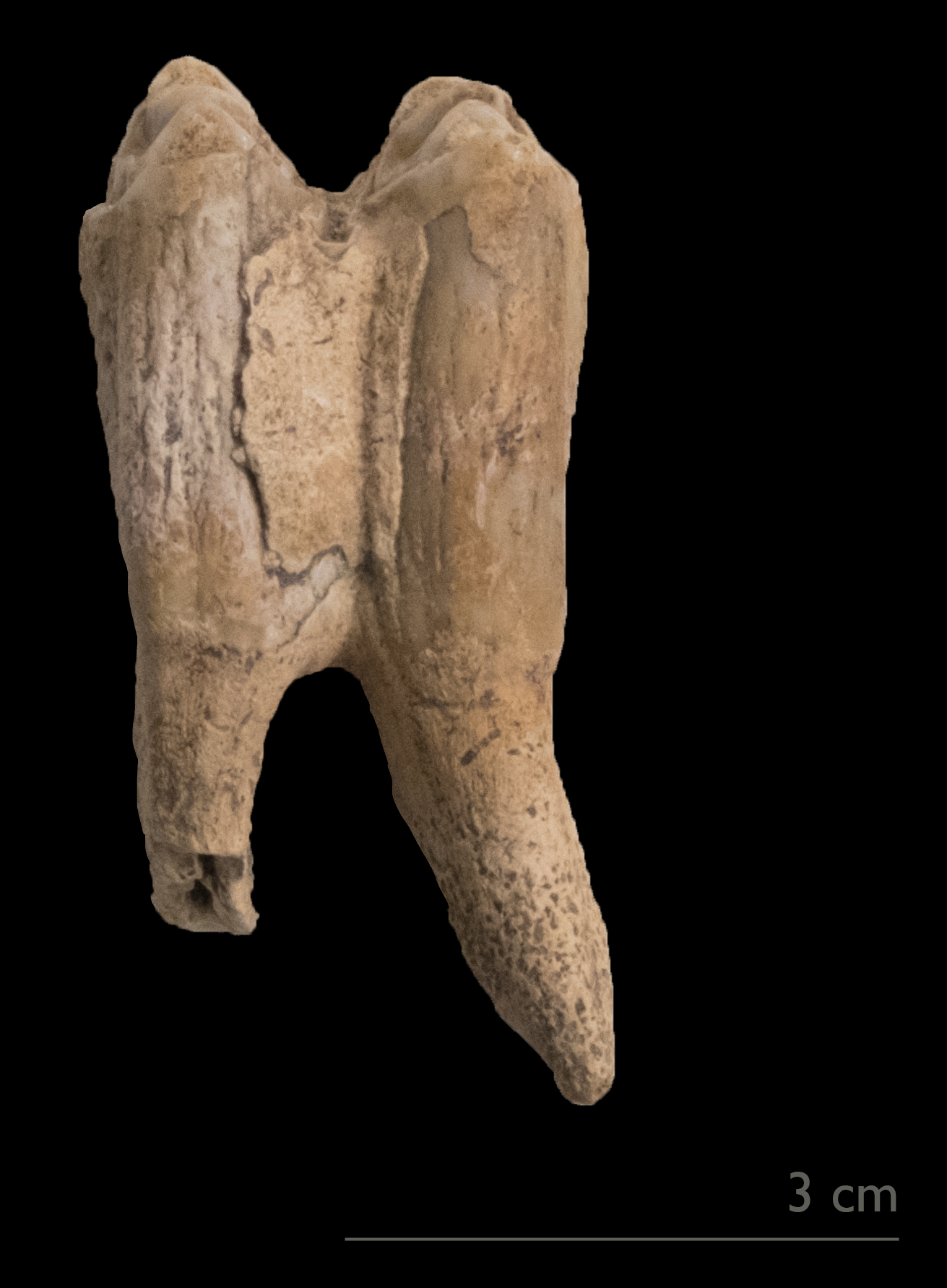

Supplement: S16 Fig — (JPG) [file pone.0213173.s017.jpg]

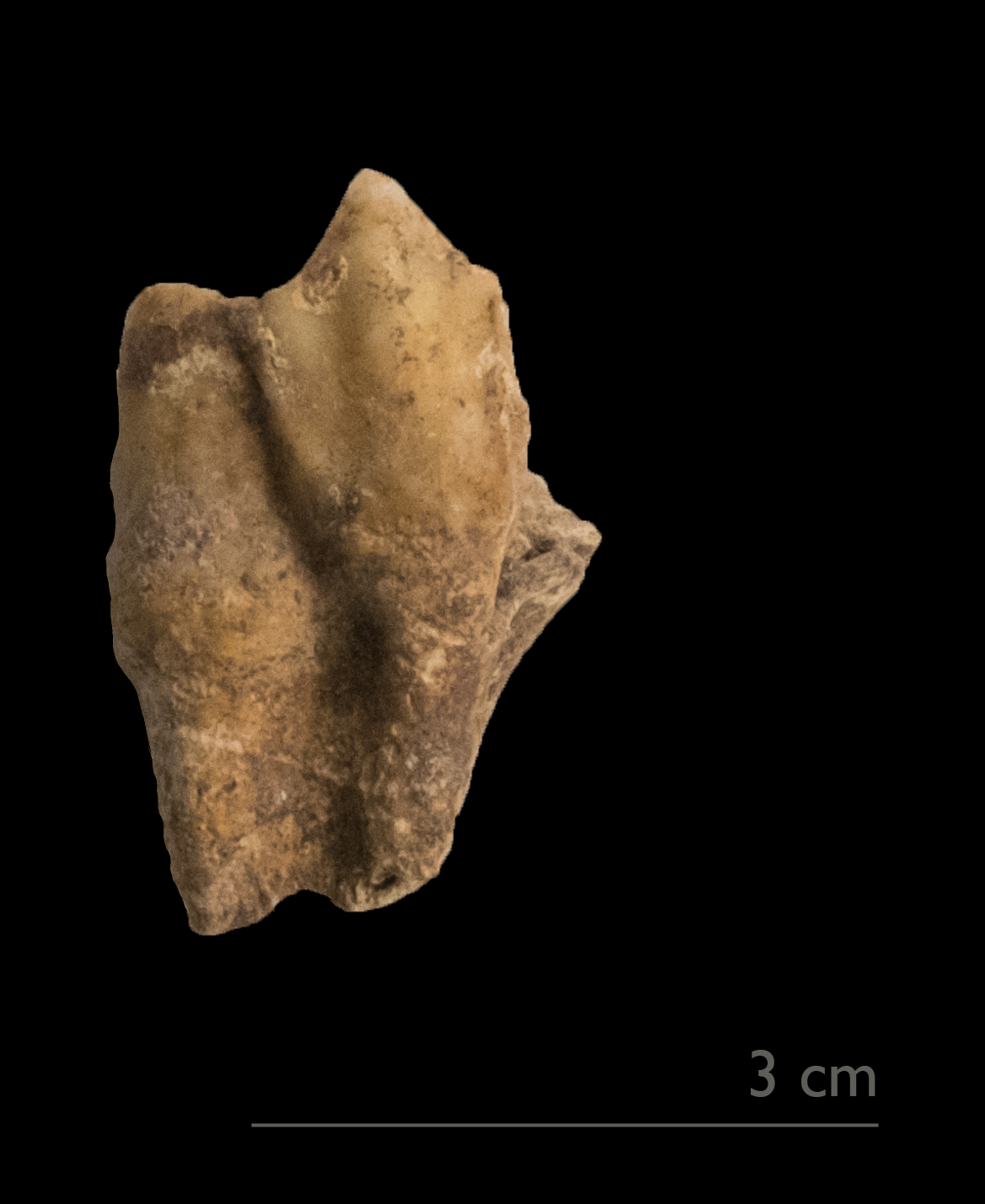

Supplement: S17 Fig — (JPG) [file pone.0213173.s018.jpg]

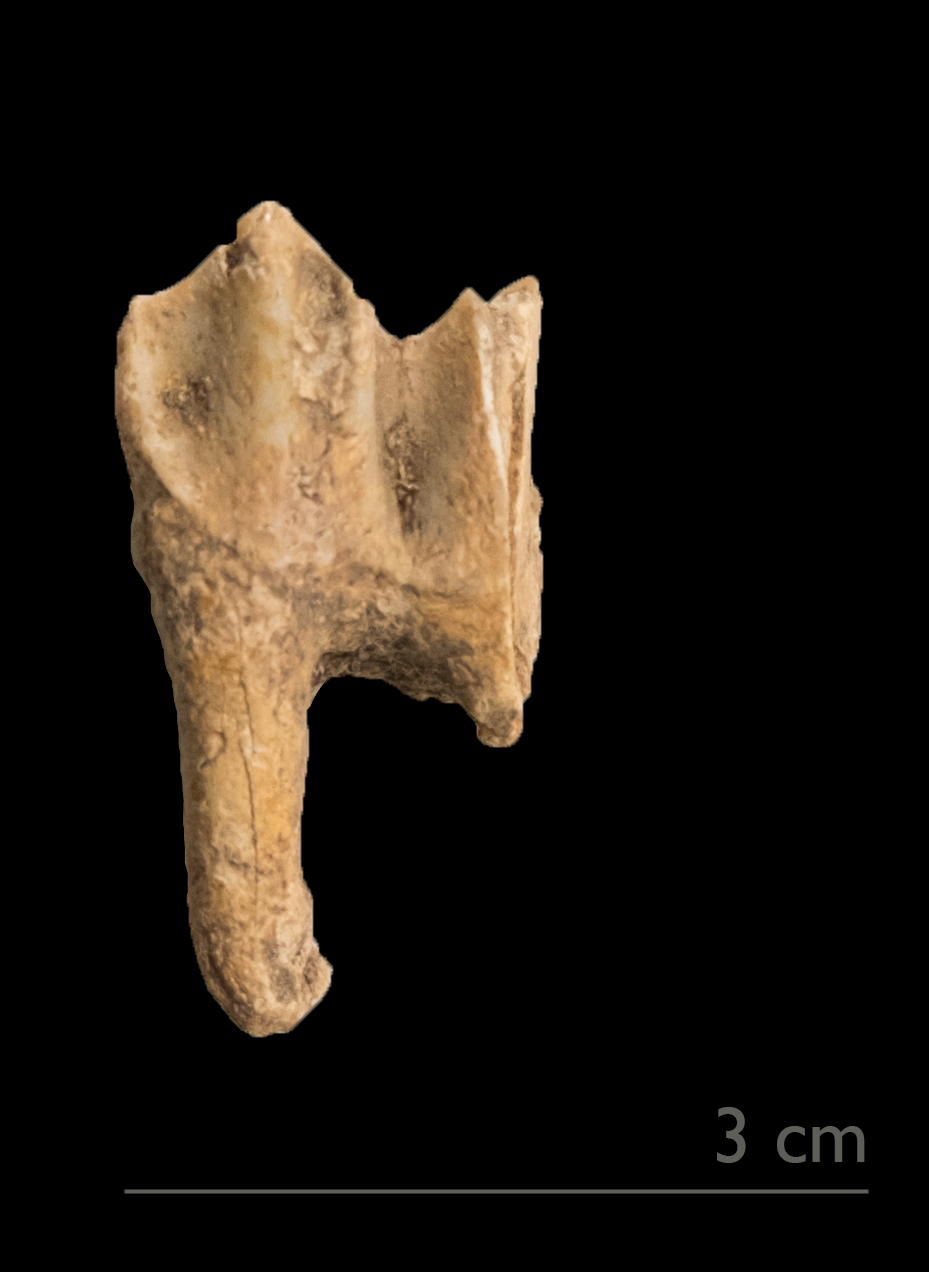

Supplement: S18 Fig — (JPG) [file pone.0213173.s019.jpg]

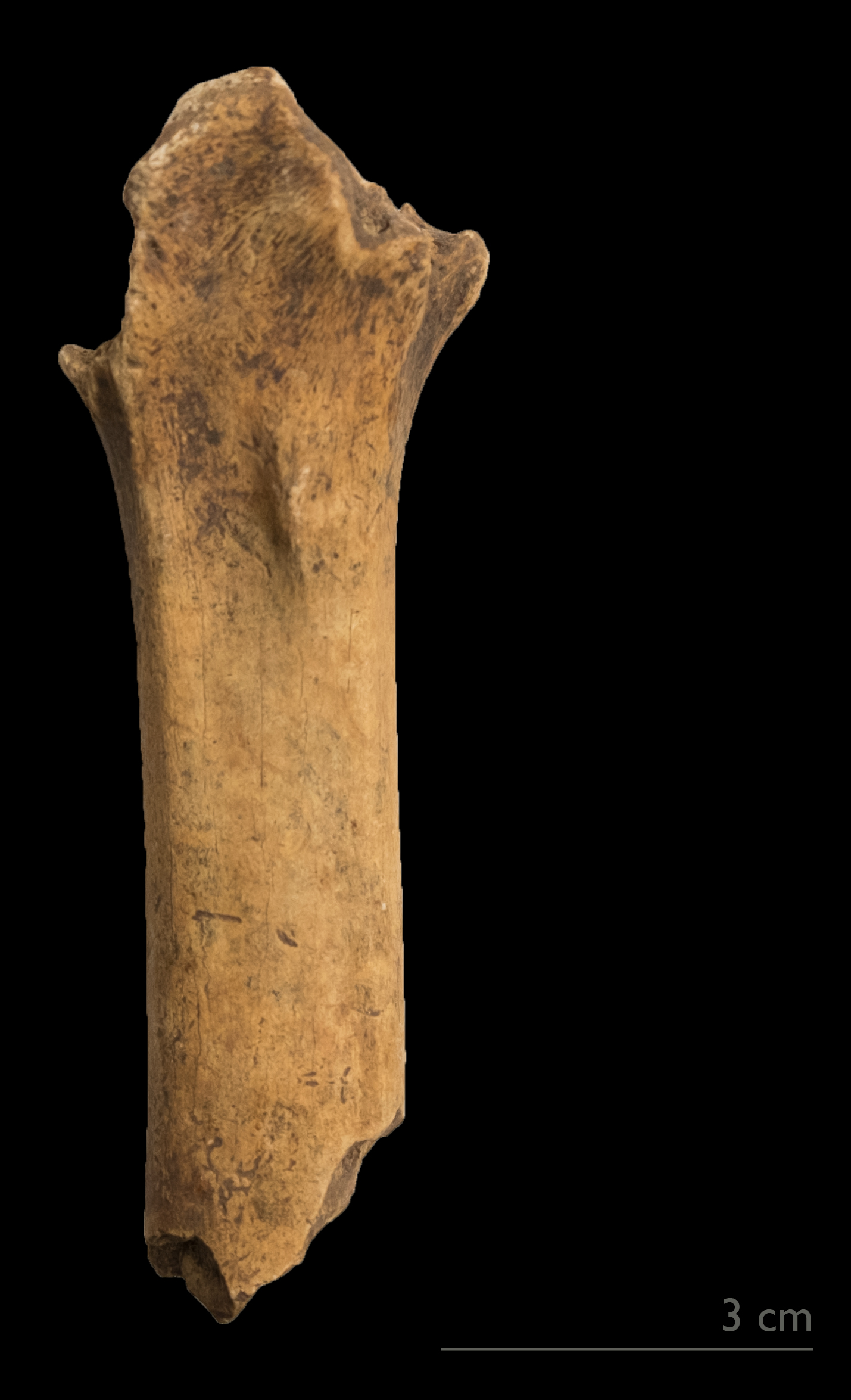

Supplement: S19 Fig — (JPG) [file pone.0213173.s020.jpg]

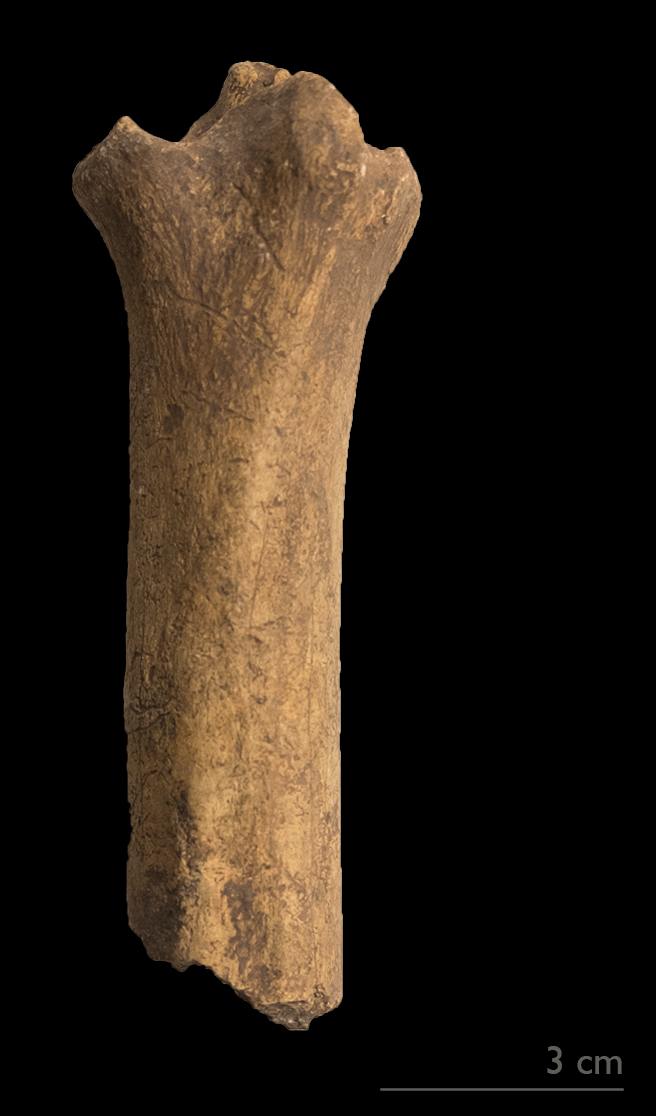

Supplement: S20 Fig — (JPG) [file pone.0213173.s021.jpg]

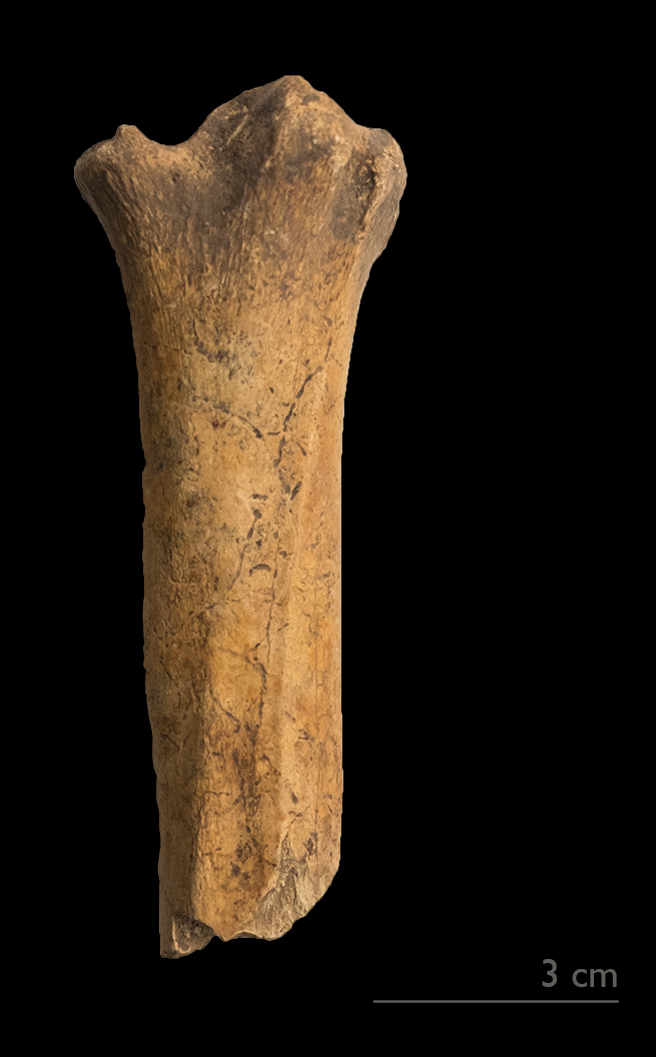

Supplement: S21 Fig — (JPG) [file pone.0213173.s022.jpg]

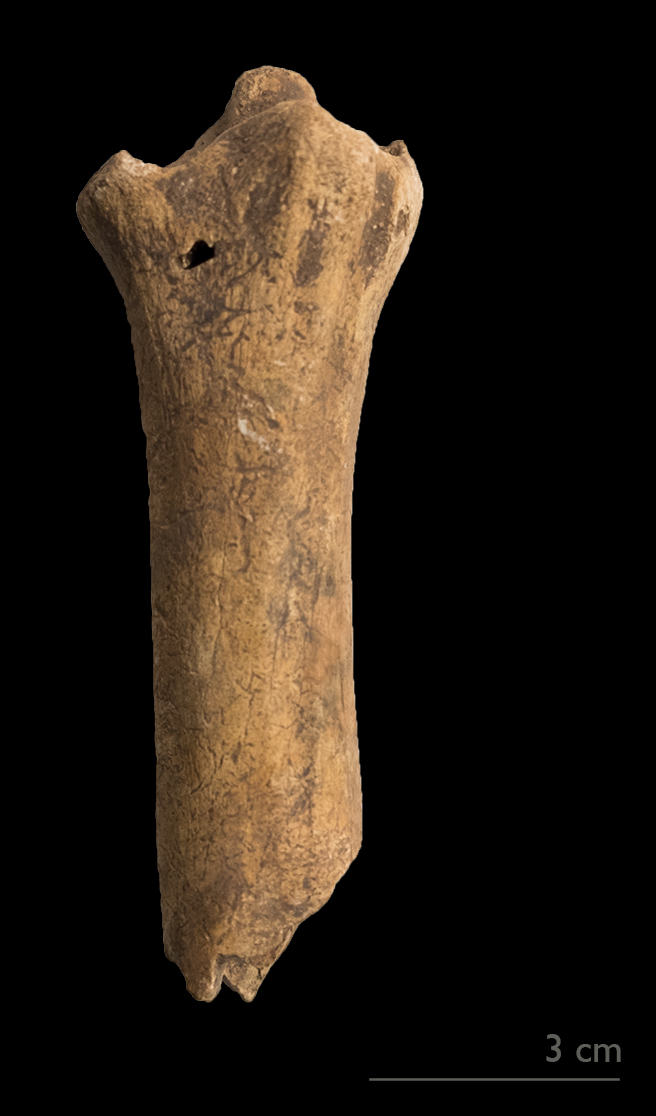

Supplement: S22 Fig — (JPG) [file pone.0213173.s023.jpg]

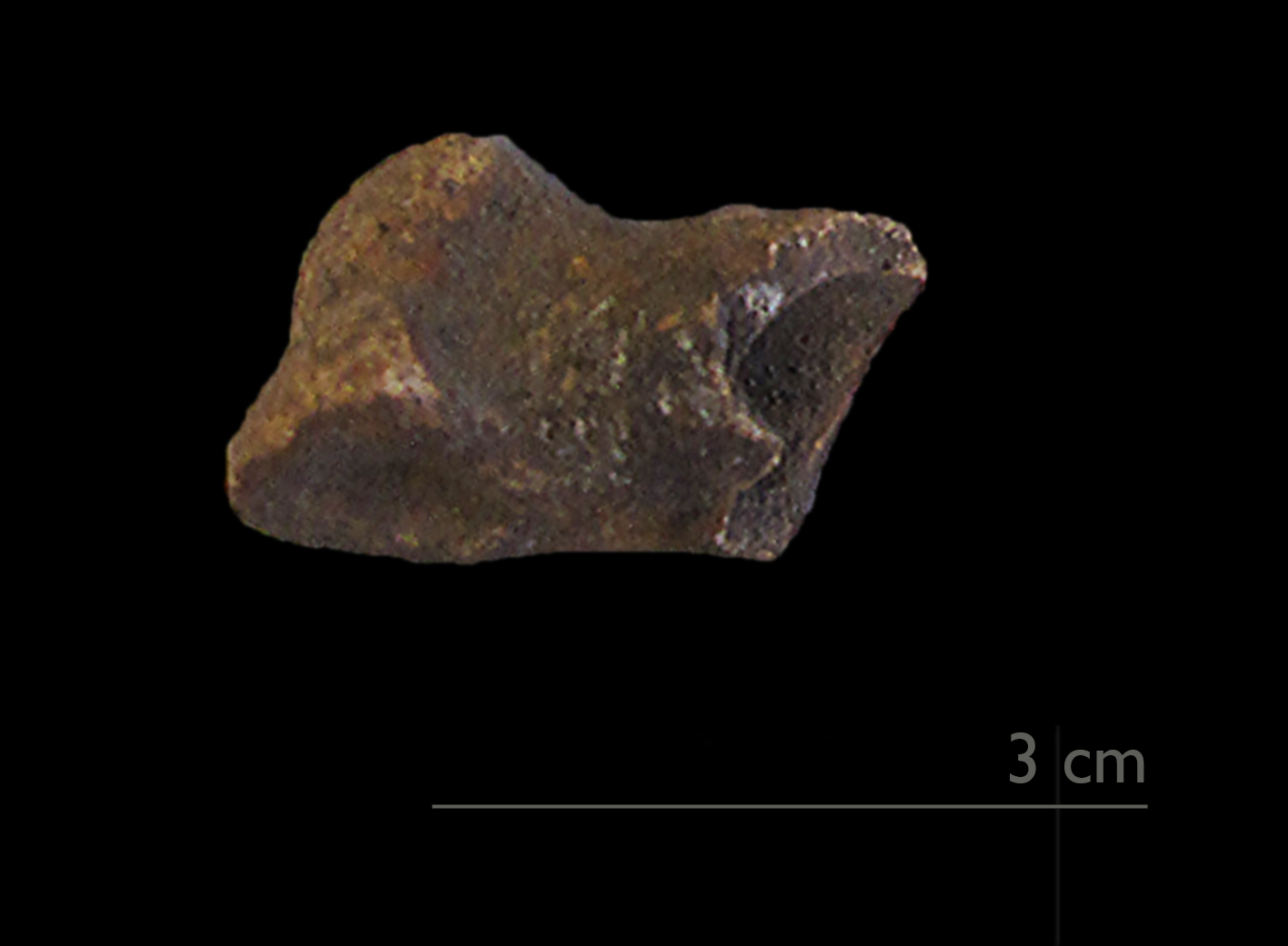

Supplement: S23 Fig — (JPG) [file pone.0213173.s024.jpg]

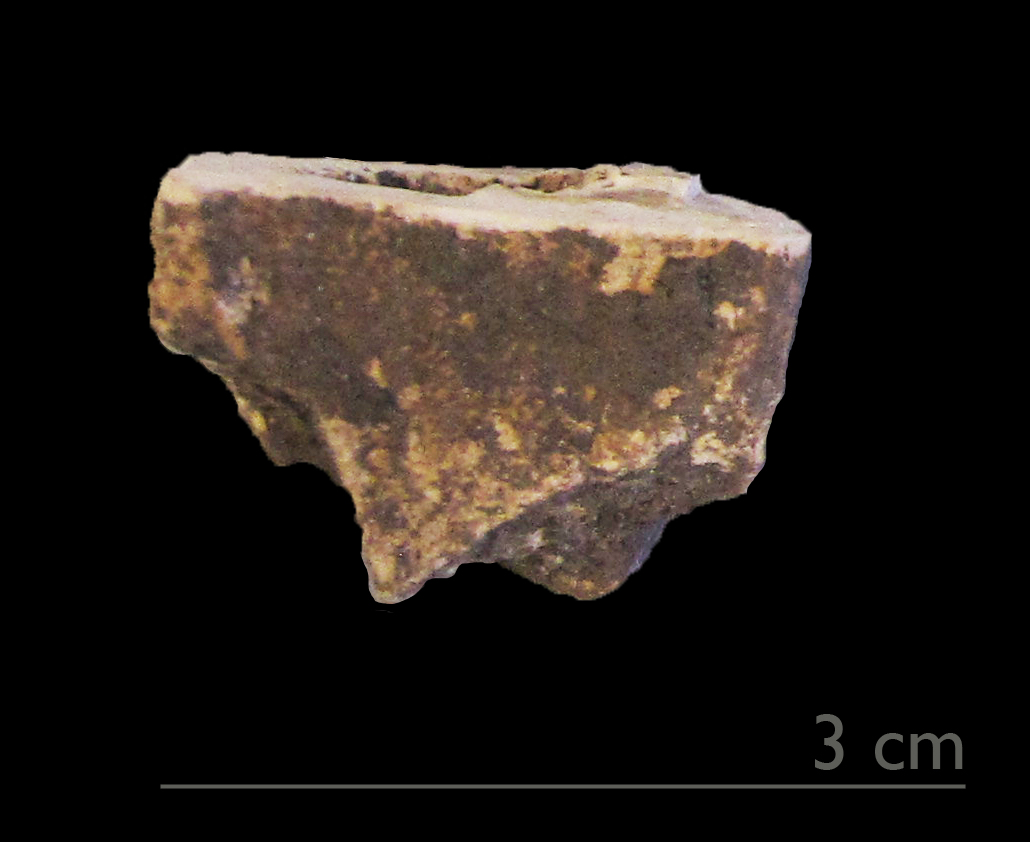

Supplement: S24 Fig — (JPG) [file pone.0213173.s025.jpg]

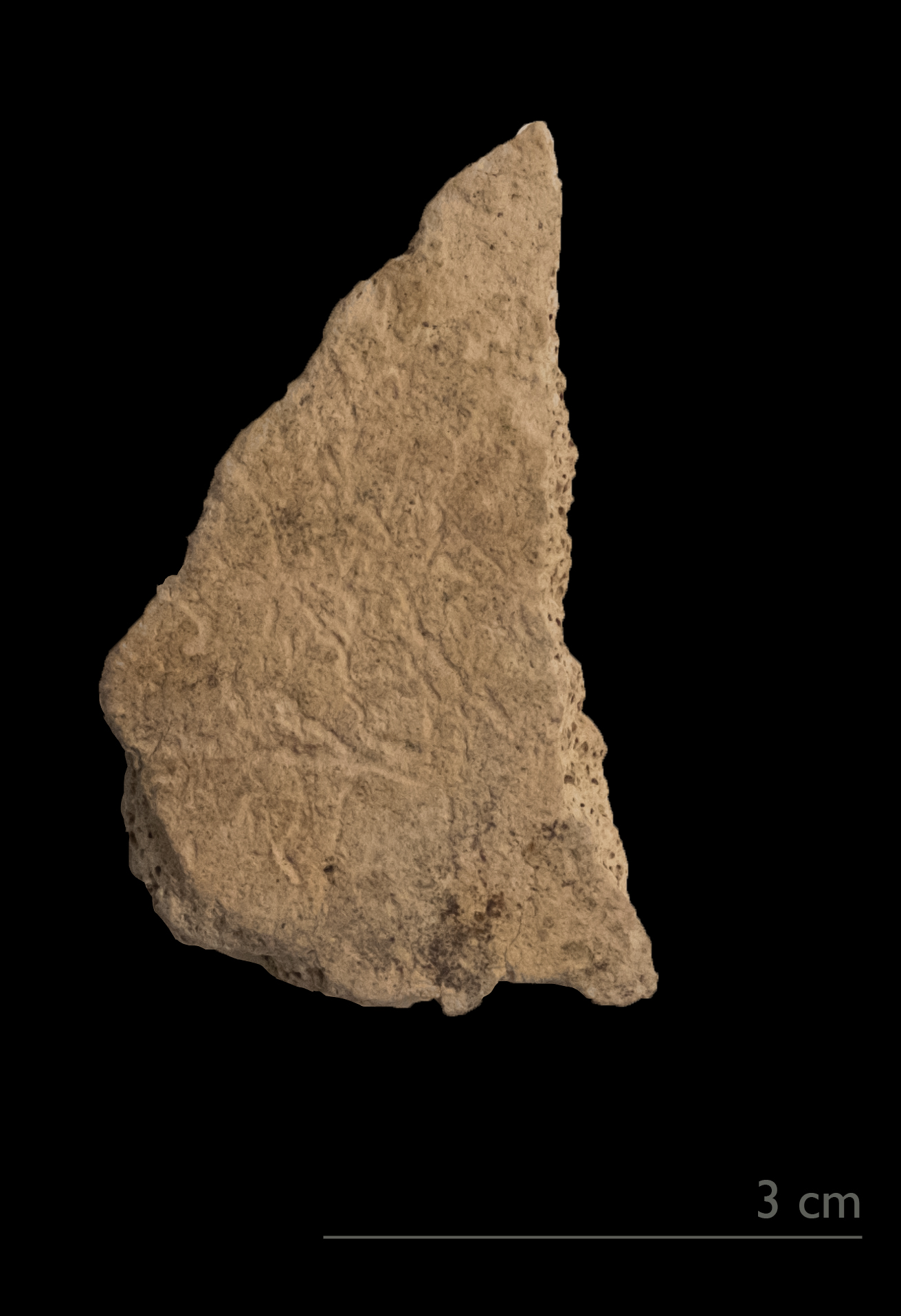

Supplement: S25 Fig — (JPG) [file pone.0213173.s026.jpg]

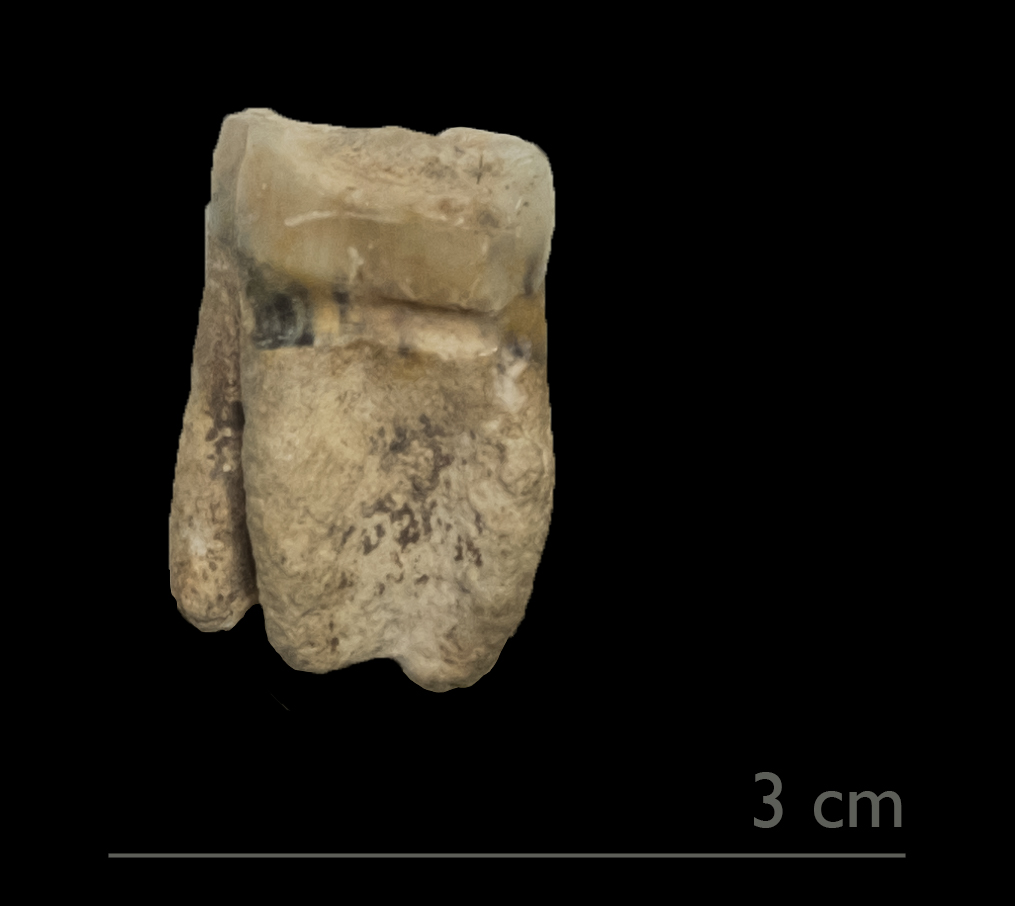

Supplement: S26 Fig — (JPG) [file pone.0213173.s027.jpg]

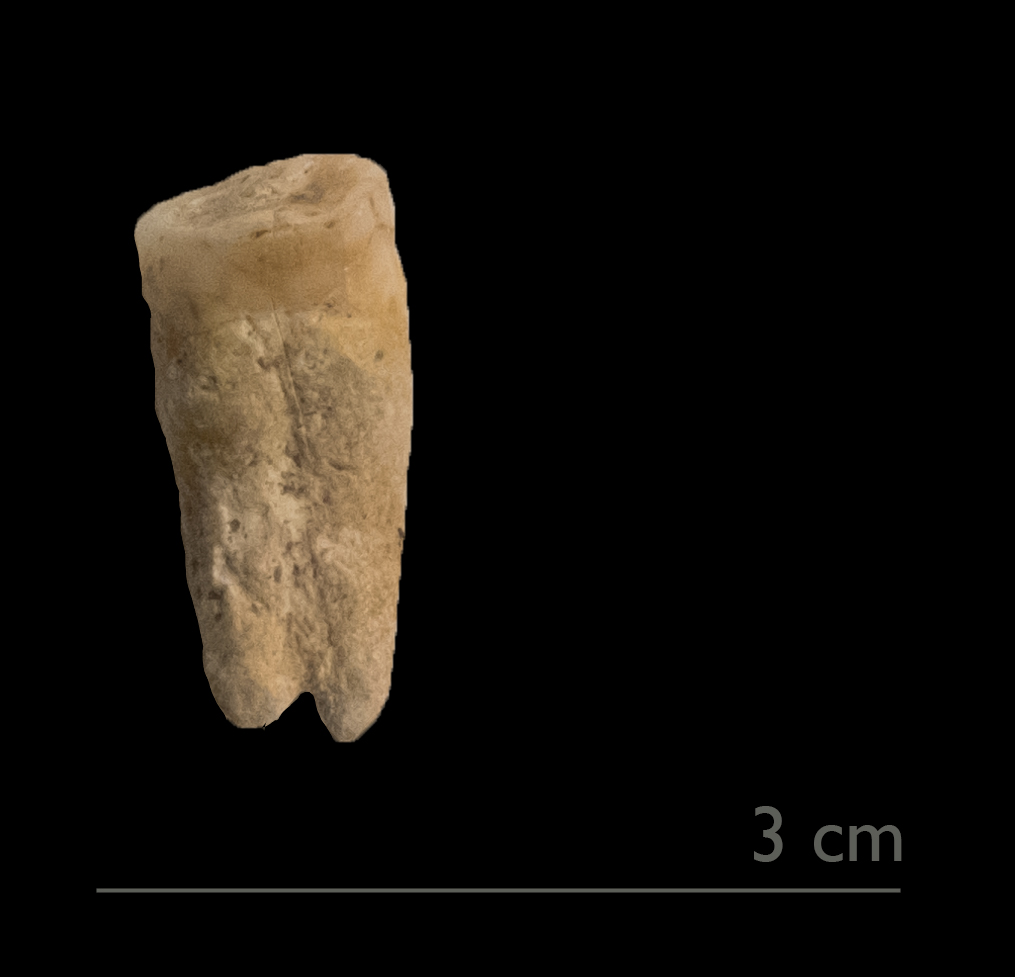

Supplement: S27 Fig — (JPG) [file pone.0213173.s028.jpg]
