# Supplementary material for: Radiocarbon dating and isotope analysis on the purported Aurignacian skeletal remains from Fontana Nuova (Ragusa, Italy)
Source: PLoS One. 2019 Mar 20;14(3):e0213173. doi: 10.1371/journal.pone.0213173 (PMC6426221; doi:10.1371/journal.pone.0213173)
Supplement: S1 Appendix — (DOCX) [file pone.0213173.s029.docx]

Plot()

{

Sequence()

{

Boundary("Start 1");

Phase("1")

{

R_Date("MAMS-30411", 8793, 22);

R_Date("MAMS-30412", 8720, 22);

R_Date("MAMS-30406", 8701, 22);

R_Date("MAMS-30405", 8699, 22);

R_Date("MAMS-30409", 8680, 21);

R_Date("MAMS-30660", 8675, 25)

{

color="red";

};

R_Date("MAMS-30663", 8658, 25)

{

color="red";

};

R_Date("MAMS-30410", 8597, 22);

R_Date("MAMS-30404", 8285, 20);

R_Date("MAMS-30407", 7775, 20);

};

Boundary("End 1");

};

};
